# Sustainable Production of Bio-Based Geraniol: Heterologous Expression of Early Terpenoid Pathway Enzymes in *Chlamydomonas reinhardtii*

**DOI:** 10.1021/acssynbio.5c00510

**Published:** 2025-08-26

**Authors:** Federico Perozeni, Edoardo Ceschi, Giovanni Luzzini, Davide Slaghenaufi, Matteo Pivato, Stefano Cazzaniga, Thomas Baier, Alexander Einhaus, Sebastian Overmans, Kyle J. Lauersen, Maurizio Ugliano, Matteo Ballottari

**Affiliations:** 1 Department of Biotechnology, 19051 University of Verona, Strada le Grazie 15, Verona 37134, Italy; 2 Faculty of Biology, Center for Biotechnology (CeBiTec), 9167 Bielefeld University, Universitätsstrasse 27, Bielefeld 33615, Germany; 3 Bioengineering Program, Biological Environmental Sciences and Engineering Division (BESE), 127355 King Abdullah University of Science and Technology (KAUST), Thuwal 239555, Saudi Arabia

**Keywords:** geraniol, *Chlamydomonas reinhardtii*, metabolic engineering, terpenes, geraniol synthase, geranyl diphosphate synthase

## Abstract

Geraniol is a monoterpene alcohol with a rose-like aroma, used in food and cosmetics and for its anti-inflammatory, antibacterial, and insect-repellent properties. Geraniol is commonly chemically synthesized from petroleum-based sources in a highly energy-demanding process with a large carbon footprint. Alternatively, geraniol can be derived from plant-based essential oils but with relatively low yields and limitations from seasonal cultivation. Here, a sustainable geraniol biosynthesis alternative was established in the photosynthetic green microalga *Chlamydomonas reinhardtii*. Three enzymes—geraniol synthase from *Catharanthus roseus* (*Cr*GES), geranyl diphosphate synthase from *Lithospermum erythrorhizon* (*Le*GPPS), and a modified 1-deoxy-d-xylulose-5-phosphate synthase from *Salvia pomifera* (*Sp*DXS)—were strategically redesigned for high expression from the algal nuclear genome. Various enzyme combinations and subcellular localizations were tested, resulting after 48 h in up to 1 mg geraniol/L (corresponding to 1.8 mg/g of dry weight) secreted into the culture medium. This work demonstrates a promising route for sustainable, CO_2_-based production of geraniol in microalgae and provides a foundation for further optimization.

## Graphical abstract

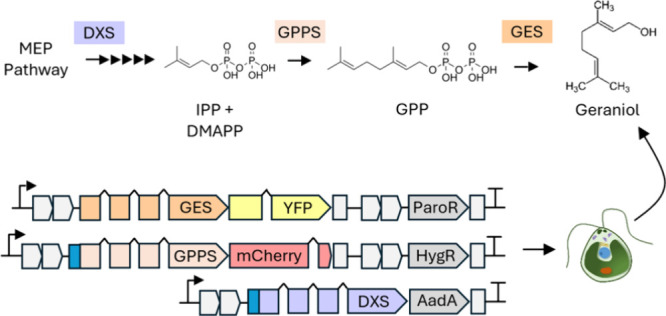

## Introduction

Terpenes make up a class of molecules produced as secondary metabolites in plants, algae, liverworts, fungi, and some insects. To date, more than 30,000 different terpene molecules have been identified. Terpenes are often scented molecules synthesized by plants to attract pollinators, repel herbivores, or attract herbivore predators. Moreover, carotenoids, the phytol chain of chlorophyll, and some phytohormones (e.g., gibberellins) are also terpene derivatives, which are required chemicals in photosynthesis and cell physiology.^1,2^ Terpene biosynthesis can occur both in the cytosol or in the plastid, and it follows two distinct pathways, the mevalonate pathway (MVA) and the methyl-erythritol-phosphate pathway (MEP), which produce the two building block molecules common to all terpenes, isopentenyl-5-diphosphate (IPP) and dimethylallyl diphosphate (DMAPP). These two pathways are mutually exclusive in most organisms, except for some bacteria and land plants.^3^ The MEP pathway is present in most microalgae genera, including the model organism *Chlamydomonas reinhardtii* (*C. reinhardtii*), where it is the only source of isopentenyl precursors IPP and DMAPP (Figure 1A).^4^ Terpenes can be classified by the number of isopentenyl group atoms in the inner skeletal structure, each one being composed of five carbon atoms (C); a terpene made of a single isopentenyl group with C_5_ is called “hemiterpene”, with two isopentenyl groups “monoterpene” (C_10_), while terpenes composed of three, four, or five isopentenyl groups are “sesqui-” (C_15_), “di-” (C_20_), and “triterpenes” (C_30_), respectively. Terpenes with a backbone >C_30_ are called “polyterpenes”. Organisms, like plants, further specialize these base isoprenoids into terpene specialty chemicals via the action of specific terpene synthases and often decorate base terpene skeletons with further functional groups through the action of enzymes like the cytochrome P450s.

**Figure 1:**
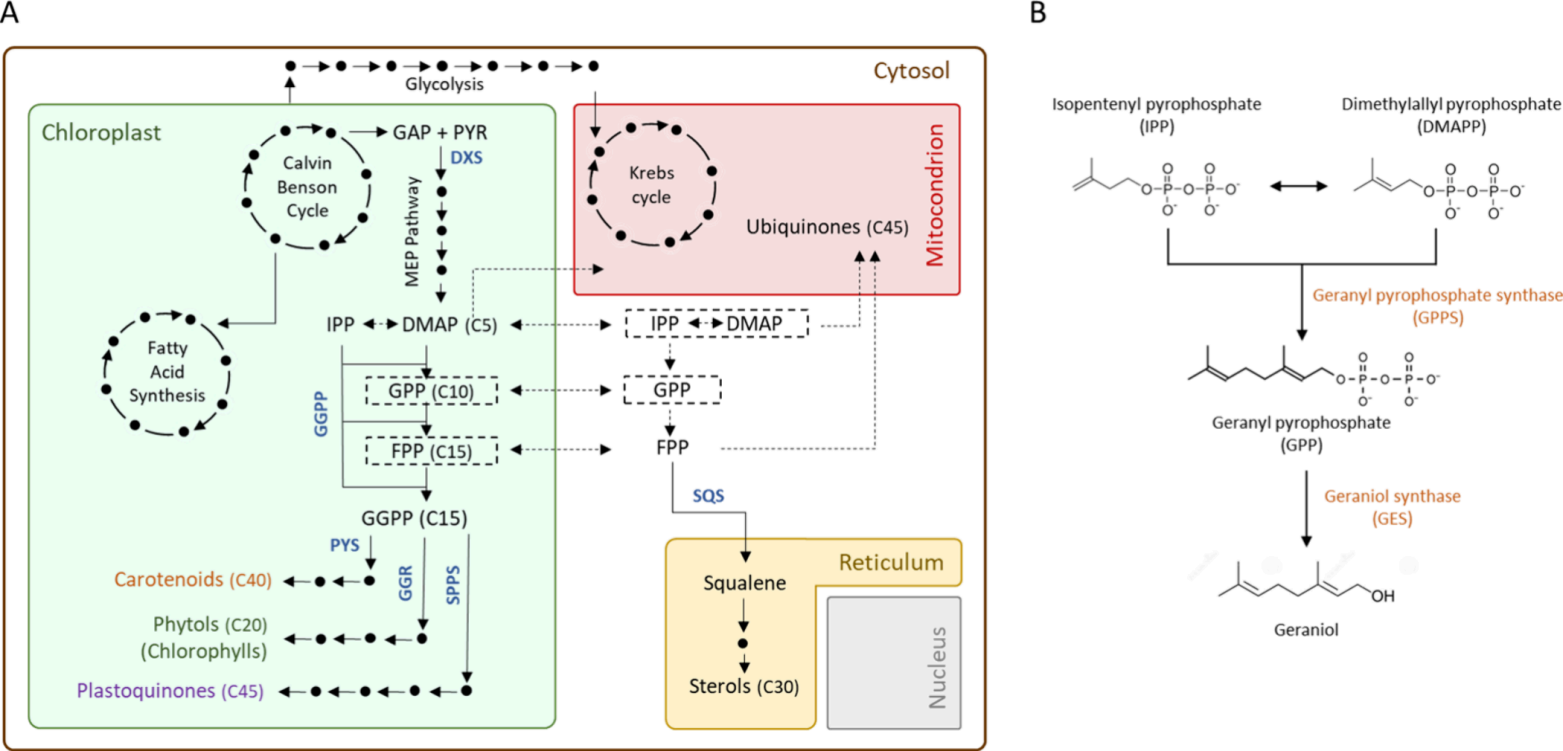
Terpenoids and geraniol biosynthetic pathways. (A) *C. reinhardtii* hypothetical terpenoid metabolism. A series of arrows represent a series of reactions; the number of arrows is not correlated to the number of reactions. Dotted squares and arrows identify metabolites or transporters whose presence is still unknown. Molecule abbreviations: PYR, pyruvate; GAP, glyceraldehyde 3-phosphate; 6PG, 6-phosphogluconate; GPP, geranyl diphosphate; FPP, farnesyl diphosphate; GGPP, geranylgeranyl diphosphate; IPP, isopentenyl diphosphate; and DMAPP, dimethylallyl diphosphate. Enzyme abbreviation (blue bold written): DXS, 1-deoxy-d-xylulose-5-phosphate synthase; GGPP, geranylgeranyl diphosphate synthase; PYS, phytoene synthase; GGR, geranyl geranyl reductase; SPPS, solanesyl diphosphate synthase; and SQS, squalene synthase. (B) Geraniol biosynthetic pathway. Enzymes are orange with their abbreviation.

The compound 3,7-dimethylocta-trans-2,6-dien-1-ol, “geraniol”, is a C_10_ monoterpene (C_10_H_18_O). The term “geraniol” relates to generally mixtures of two cis/trans isomers, with geraniol the trans form and nerol the cis. Geraniol has many interesting properties currently exploited in many areas, such as insecticide and repellent effects, antimicrobial effects, antioxidant effects, anticancer activity, and flavor.^5^ Geraniol is usually found in some plant essential oils, including *Monarda fistulosa* (70–85%), ninde (66%), rose (44%), palmarosa (∼50%), and citronella (∼25%).^6^ Geraniol is a molecule of commercial interest whose market reached US-$ 12 Bn in 2020 with an estimated compound annual growth rate (CAGR) of 7% (www.maximizemarketresearch.com), and its annual production was reported to exceed 1000 tons/year in 2008.^7^ Geraniol is commonly used in personal care cosmetics to impart fragrance and flavors. Besides its pleasant odor, geraniol exhibits antimicrobial and repellent properties with low toxicity to humans, allowing it to be used as a natural protective agent against insects and microorganisms. Geraniol is Generally Recognized As Safe (GRAS) by the Food and Drug Administration (FDA, 21 CFR 182.60) and Flavor and Extract Manufacturers’ Association (FEDA).^7^

Geraniol is most commonly produced by chemical synthesis through routes such as the reduction of citral (Grignard reaction using geranyl chloride), the hydration of myrcene (produced from pinene), or the selective hydrogenation of geranial.^8^ These methods are energy demanding and rely on petrochemical intermediates like isoprene or pinene-derived myrcene with a relatively high carbon footprint.^8^ Only a small amount of geraniol is produced by extraction from plant essential oils, mainly citronella and palmarosa oil,^8^ but due to seasonal cultivation and environmental changes, the constant supply natural geraniol is limited. Biological production of geraniol requires the catalytic activity of geraniol synthase (GES) (Figure 1B). The geraniol synthase (GES) catalyzes the formation of geraniol from geranyl diphosphate (GPP) through an ionization-dependent mechanism. The respective substrate GPP is a common metabolite in land plants and microorganisms, while GES is present only in some land plants.^9^

Expression of terpene synthases in heterologous host systems can enable the conversion of cellular intermediates into chemical products of the synthase. This is the basis of metabolic engineering efforts and has become an established technology.^10^ Recombinant expression of GES from *Ociumum basilicum* (*O. basilicum*) in *Escherichia coli* (*E. coli*) resulted in 2 g/L in a controlled fermentation process.^11^ Moreover, the fusion of GES from *O. basilicum* with the GPPS from *Abies grandis* allowed subtle improvement to 2.1 g/L.^12^

Recombinant expression of GES in *Saccharomyces cerevisiae* (*S. cerevisiae*) and optimization of its MVA pathway resulted in geraniol titers of 36 mg/L.^13^ The need to further improve geraniol production led to testing several GES in yeast, ranking their metabolic activity.^14^ The best-performing GES tested in *S. cerevisiae* was that from *Catharanthus roseus* (*C. roseus*), with an accumulation of ∼43 mg/L without further modification. By protein-directed evolution based on structure analysis and modeling of *C. roseus* GES (CrGES) and farnesyl diphosphate synthase (Erg20) using *S. cerevisiae* already overexpressing the truncated 3-hydroxy-3-methylglutaryl-coenzyme reductase (tHMGR) and the isopentenyl diphosphate isomerase (IDI1), a maximum geraniol concentration of 1.68 g/L in fed-batch conditions was achieved using ethanol as a carbon restriction strategy. To date, this is the highest geraniol concentration reported from eukaryotic host cells.^14^ Even if heterotrophic cultivation allows high production yields, producing geraniol photoautotrophically is preferable for its sustainability and lower environmental impact of algal cultivation compared to traditional fermentation. For example, the diatom *Phaeodactylum tricornutum* (*P. tricornutum*) was recently engineered for a constitutive expression of *Cr*GES, which produced up to 300 μg/L of geraniol.^15^

To give an alternative for sustainable production, we genetically engineered the freshwater model microalga *C. reinhardtii*. Efficient production of terpenes in *C. reinhardtii* has indeed recently been reported, taking advantage of advanced synthetic biology tools already available for this host.^16–22^ In this work, geraniol biosynthesis was achieved by the expression of several key enzymes for the synthesis of geraniol and its precursors. The geraniol molecule produced in *C. reinhardtii* was mainly released into the growth medium, which may simplify its harvest. By employing the heterologous expression of key terpenoid pathway enzymes in *C. reinhardtii*, it has become possible to achieve a sustainable, bio-based production of geraniol, offering a potential alternative to conventional methods for microbial terpenoid synthesis.

## Results and Discussion

### CrGES Expression in *C. reinhardtii* Results in Geraniol Accumulation

In this study, we tested the *C. roseus* GES in the *C. reinhardtii* UVM4 strain, a mutant strain generated by random mutagenesis and selected for enabling more reliable transgene expression from the nuclear genome.^23^ A synthetic sequence encoding *C. roseus* GES (hereafter referred to as *Cr*GES) was designed according to the strategy previously described for *C. reinhardtii*.^19,24^ Briefly, the *Cr*GES nucleotide sequence was codon-optimized for *C. reinhardtii*, and three copies of the first intron from the ribulose-1,5-bisphosphate carboxylase/oxygenase small subunit 2 (RBCS2) were inserted at a specific frequency. The gene was cloned upstream of the mVenus (yellow fluorescent protein, YFP) sequence in a pOptimized 2 plasmid, in which the YFP contains the second rbcs2 intron sequence. Fusion of the *Cr*GES to the fluorescent protein was used to enable screening of transformant lines on the basis of fluorescence. No additional target peptide was added to the GES as it natively contains one from its progenitor plant.^25^ The synthetic *Cr*GES coding sequence was placed under the control of the hybrid promoter HSP70/RBCS, a previously described strong promoter for *C. reinhardtii*.^26^ A second expression cassette with paromomycin resistance (APHVIII) was also used as a selection marker. All these strategies are commonly used to increase gene expression in *C. reinhardtii*.^16^ After transforming the UVM4 strain with the assembled vector, colonies grown in the presence of the selection agent (paromomycin) were then screened for YFP emission (Supplementary Figure 1). Only a few of these selected lines showed fluorescence values higher than the average because of random insertions into the algal genome. Putative expressing lines with the highest fluorescence were analyzed by Western blotting using an anti-GFP antibody. UVM4 was also tested as a negative control (Supplementary Figure 1, insert). Only three lines, F4, A11, and G2, among the selected lines were characterized by a clear band, which resulted in an apparent molecular weight of 75 kDa. The expected molecular weight of *Cr*GES_YFP was 92 kDa; the 75 kDa reported probably resulted from removal of the plastid targeting peptide or proteolysis. Gas Chromatography–Mass Spectrometry (GC-MS) analysis confirmed the presence of geraniol in the three *Cr*GES accumulating lines. Geraniol was detected as a peak in GC-MS chromatograms (Figure 2A and Supplementary Figure 2), with a retention time of 31.2 min and the expected mass fractionation pattern, thereby confirming the catalytic activity of the recombinant *Cr*GES expressed in *C. reinhardtii*. No geraniol peak was detected in chromatograms of the parental strain UVM4 samples. Cellular pellets and growth media were analyzed, and 92.32% of the total geraniol was detected in the culture supernatant, while only a small part remained in the pellet (Figure 2B). The preferential distribution of geraniol in the liquid phase is due to its volatility, a common feature of monoterpenes, which passively diffuse from cells to a culture medium.^20,27–30^ Geraniol production was also analyzed in *Cr*GES expressing lines over time (Figure 2C). The maximum geraniol accumulation in the culture medium was detected 48 h after the inoculation followed by a strong product decrease with a reduction of 50% after 72 h and only small amounts detectable after 96 h. The geraniol loss could be due to product inhibition of the *Cr*GES enzyme or time-dependent protein abundance, which was evaluated by monitoring YFP fluorescence during growth. However, YFP fluorescence showed no significant reduction over time, suggesting stable expression of *Cr*GES (Supplementary Figure 3). This result is expected considering the use of the HSP70-RBCS2 constitutive promoter, which is well known for its ability to drive high-level gene expression constitutively.^26^ Possibly, *Cr*GES catalytic activity could be inhibited by its product but potential reduced *Cr*GES activity cannot explain the disappearance of the already produced geraniol.

**Figure 2:**
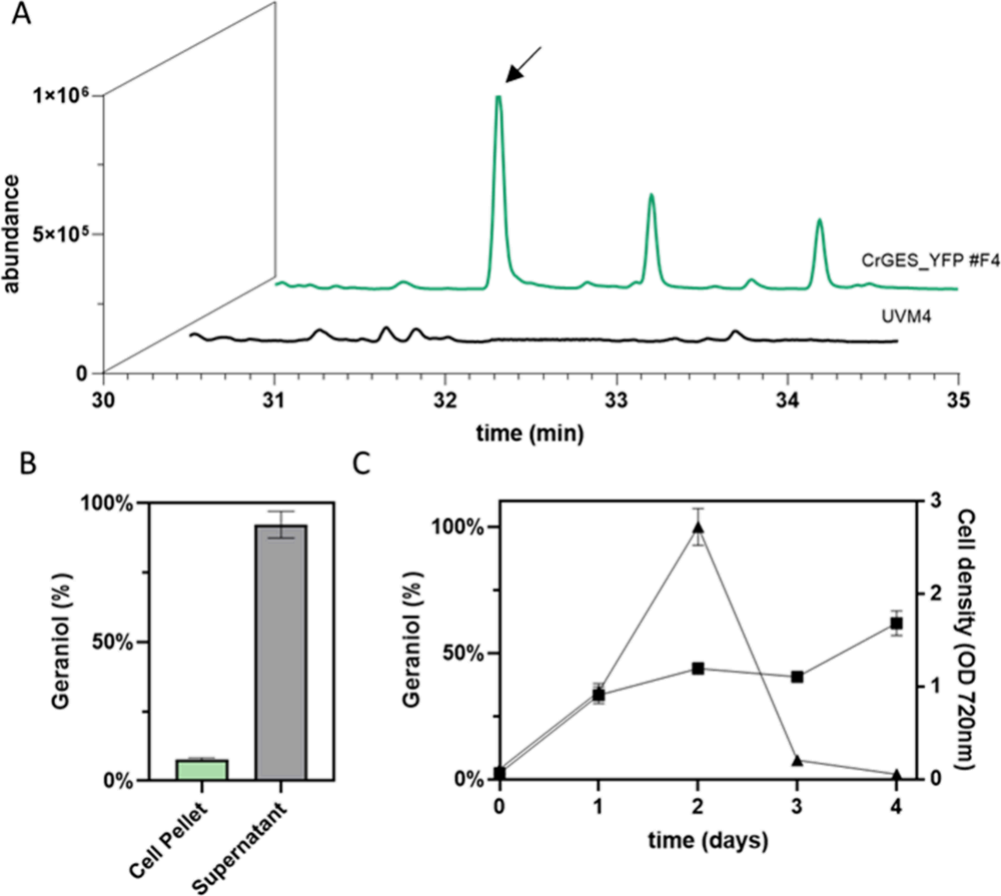
Analysis of geraniol production in engineered lines. (A) Geraniol (retention time of 31.2 min, black arrow) detection by GC-MS in *Cr*GES_YFP expressing line F4; no peak was detectable in the parental strain (UVM4). (B) Distribution of geraniol inside or outside the *Chlamydomonas* cells. Analysis was made as described in the Materials and Methods section. (C) Geraniol accumulation kinetics (triangles) and cell density (squares) of the *Cr*GES_YFP F4 expressing line. Only the cultivation medium was sampled.

Evaporation or degradation can be thus hypothesized according to what was observed in *E. coli*.^31^ To investigate the evaporation tendency of geraniol and/or a possible geraniol-degrading activity by *C. reinhardtii* cells, commercial geraniol was added to the TAP medium in the presence or absence of the *C. reinhardtii* UVM4 strain. Data reported in Supplementary Figure 4 reveal that, in the absence of *C. reinhardtii* cells, ∼40% of total geraniol was released by evaporation after 72 h, contributing to the disappearance measured in the engineered geraniol-producing strain after 2 days of cultivation. Besides, a faster and more abundant decrease in geraniol content was recorded when a geraniol standard was added in the presence of UVM4 cells. Indeed, the presence of *C. reinhardtii* cells led to the disappearance of ∼80% of total geraniol after 12 h, while no traces of this molecule could be detected after 72 h of cultivation. This result shows that geraniol was actively degraded by algae-mediated mechanisms of chemical interaction with cellular components. A decrease in geraniol concentration in *E. coli*-engineered strains was already reported, and the action of endogenous enzymes able to use geraniol to produce nerol and citronellol was demonstrated.^31^ We, therefore, investigated the presence of geraniol as well as nerol, citronellol, and linalool (Supplementary Figure 5). Our results show that nerol, linalool, and citronellol were detected with a slight increase after 3 days of cultivation, the total terpene amount was strongly reduced. Nerol, citronellol, and linalool were thus side products of *Cr*GES activity instead of being actively converted; the presence of side products is a common feature of terpene synthases.^32,33^ However, we cannot exclude the conversion of geraniol by *C. reinhardtii* metabolism in other metabolites not detected in our analysis. Additional work is required to identify possible degradation products of geraniol and, thus, design additional metabolic engineering to inhibit these competing metabolic reactions.

Quantification of geraniol from the supernatant revealed a mean value of 42.2 μg/L for *Cr*GES_YFP expressing lines (Figure 3, first line) with the F4 line being the best performing with an accumulation of up to 87 μg/L. It is interesting to note that *Cr*GES_YFP expressing lines showed a linear correlation between geraniol and GES expression (Supplementary Figure 6).

**Figure 3:**
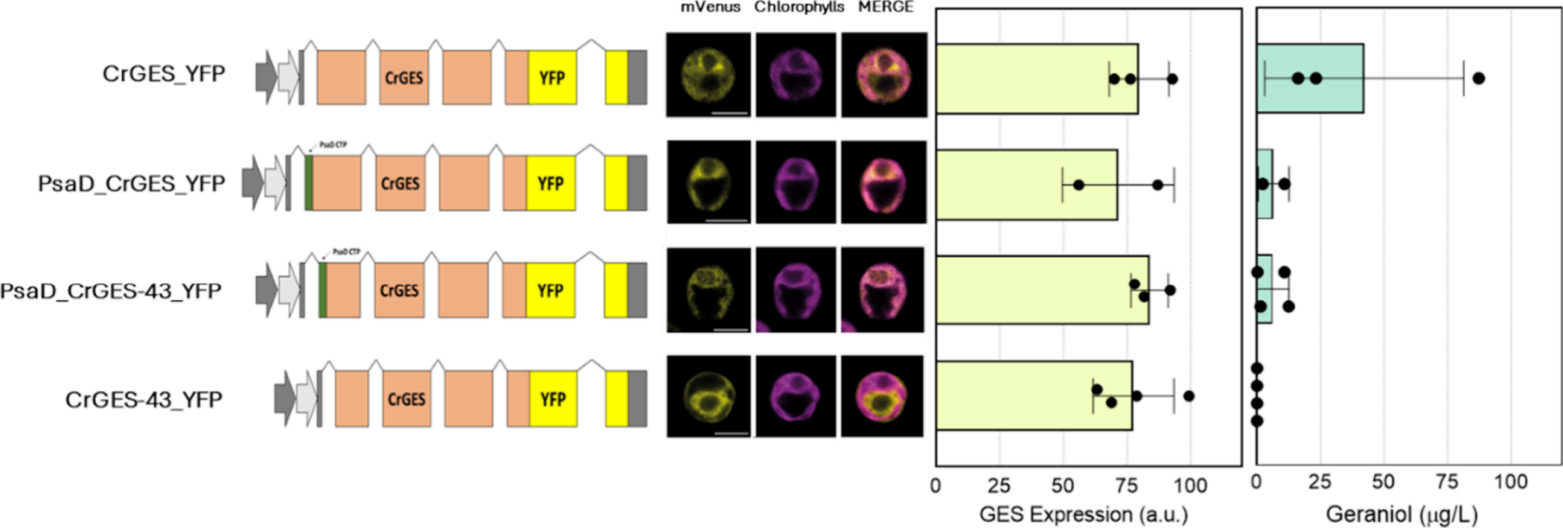
Vector diagram, localization, *Cr*GES expression level, and geraniol accumulation of all of the *Cr*GES expressing lines generated in this work. For localization, YFP fluorescence (mVenus), chlorophyll autofluorescence, and the merger of these two channels are shown. Excitation for YFP was 514 nm and 633 nm for chlorophylls. Emission was detected at 522–572 nm for YFP and 670–690 nm for chlorophylls. The scale bar represents 5 μm. The GES expression level is based on YFP fluorescence of selected lines, considering the equimolar ratio between GES and YFP. *Cr*GES detection was performed only in the cultivation medium. Dots in *Cr*GES expression and geraniol accumulation represent the average of three technical replicates for individual lines.

### Localization of CrGES Is Crucial for Geraniol Production

The isoprenoid metabolism of *C. reinhardtii* differs significantly from that of higher plants: land plants have both the MEP (chloroplast) and the MVA pathway (cytosol) to synthesize C_5_ IPP and DMAPP, whereas *Chlorophyceae* possess only the MEP pathway^4^ (Figure 1A). Synthesis of farnesyl diphosphate (FPP) was reported in the cytosol, implying the availability of a cytosolic GPP/IPP and DMAPP pool or export from the chloroplast. Nevertheless, the MEP localization in the chloroplast, as well as the presence of C_10_ geranyl diphosphate (GPP) in the chloroplast, suggests that targeting the *Cr*GES enzyme to the plastid could potentially result in higher availability of precursors for geraniol synthesis compared to the cytosolic localization. *Cr*GES has been reported to contain a plastid transit peptide of 43 amino acids;^25^ the removal of which is crucial for the increasing accumulation and activity in *S. cerevisiae* *.* ^14^ In *C. reinhardtii*, expression of the *Cr*GES gene with an endogenous target peptide resulted in protein accumulation in the chloroplast, as shown in Figure 3 (first line). The YFP signal of *Cr*GES_YFP overlaps with the chlorophyll signal, confirming the presence of the protein in the plastid and the ability of the native transit peptide to direct protein into this compartment also in *C. reinhardtii*. It is essential to underline that the possibility of producing geraniol with a chloroplastic *Cr*GES confirms the presence of a GPP pool in the chloroplast, which could be directly produced by GPP synthase or released by FPP or GGPP synthase as previously observed in limonene production engineering.^34^

The PSAD transit peptide was then used to direct the *Cr*GES protein into the chloroplast as previously reported for other enzymes.^16^ The N-terminal region of PSAD was added to or used to replace the native 43 aa CrGES transit peptide. Lines were screened for fluorescence, and protein expression was confirmed by Western blotting (Supplementary Figures 7 and 8). Confocal microscopy, using YFP-tagged proteins, confirmed the localization patterns. The native 43 aa peptide directed the protein to the chloroplast, but not exclusively—some signal appeared in the cytosol–chloroplast interface. In contrast, the PSAD transit peptide enabled precise chloroplast targeting, regardless of the presence of the CrGES N-terminus (Figure 3). Surprisingly, the addition of the PSAD transit peptide at the N-terminus or its use to substitute the endogenous transit peptide caused a decreased geraniol accumulation, despite a similar protein content compared to GES expressing lines (Figure 3, second and third lines). These data suggest a possible consequence in terms of geraniol production due to a different chloroplast localization efficiency by PSAD or endogenous transit peptides or the involvement of N-terminal 43 aa in correct protein folding. Removing the N-terminal 43 amino acids (Supplementary Figure 9) resulted in cytosol localization, as shown in Figure 3, with an evident absence of overlap of the YFP and chlorophyll signals. According to the lack of C_10_ precursors in the cytosol, no geraniol was detected in any tested lines, although the *Cr*GES accumulation was comparable with other *Cr*GES-expressing genotypes (Figure 3).

The ability of the *Cr*GES N-terminal region to import proteins into the *C. reinhardtii* chloroplast was investigated by generating lines in which YFP was fused at the N-terminus with different portions of the *Cr*GES N-terminal portions, specifically from 43 aa to 100 aa (Supplementary Figure 10). The GES N-terminal 43 aa was not sufficient to direct YFP into the chloroplast, which was instead localized in vesicles at the cytosol–chloroplast interface (Figure 4). The same behavior was observed when the *Cr*GES N-terminal portion fused to YFP was extended to 60 aa. Conversely, *Cr*GES N-terminal 100 amino acids were able to localize the fluorescent protein into the chloroplast. The longer transit peptide recognized by *C. reinhardtii* for the import of *Cr*GES in the chloroplast is consistent with the Western blot analysis described herein in which the apparent molecular weight for *Cr*GES_YFP is lower than the expected one (Supplementary Figure 1). This result is in line with previous findings of the recognition of longer transit peptide by the *C. reinhardtii* TIC-TOC system compared to the land plant equivalent import system.^35,36^

**Figure 4:**
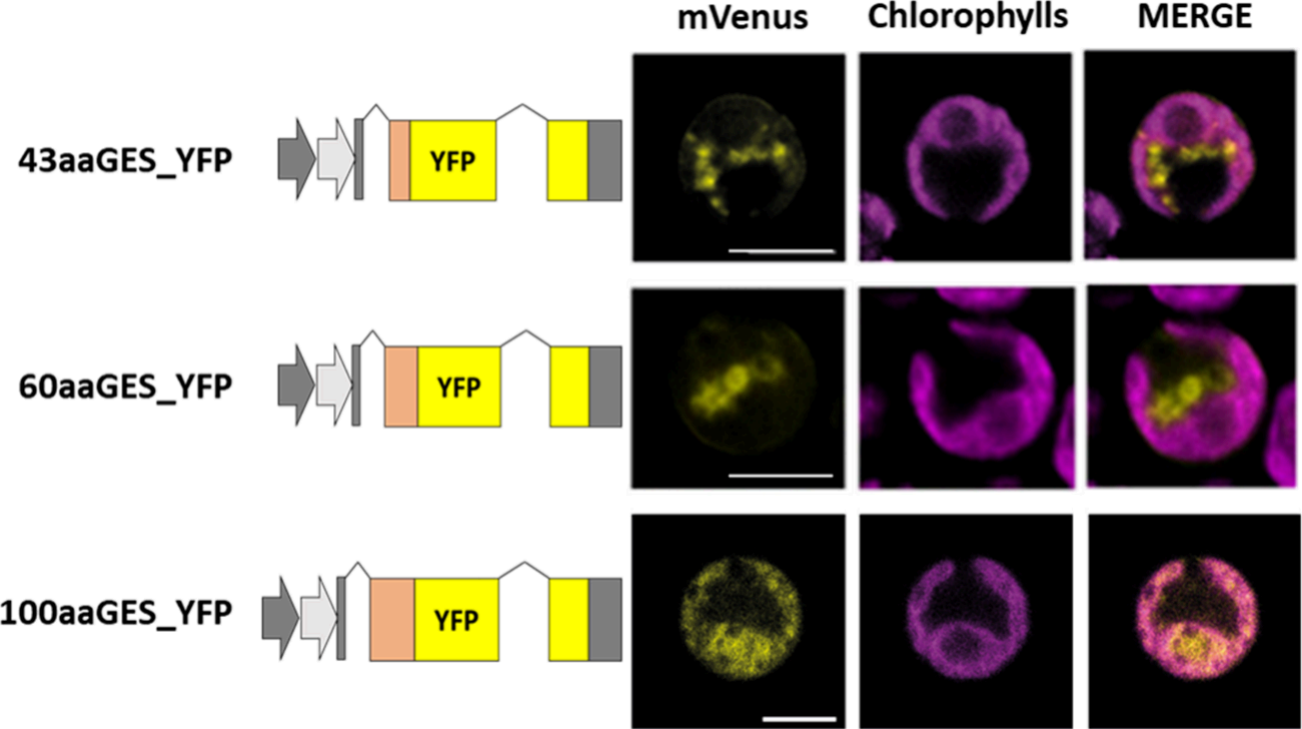
Localization of the YFP protein using putative *Cr*GES target peptide from 43 aa to 100 aa. YFP fluorescence (mVenus), chlorophyll autofluorescence, and the merger of these two channels are shown. Excitation for YFP was 514 nm and 633 nm for chlorophylls. Emission was detected at 522–572 nm for YFP and 670–690 nm for chlorophylls. The scale bar represents 5 μm.

### Accumulation of Geraniol Does Not Interfere with Algal Growth, Preserving the Biosynthesis of Photosynthetic Pigments

Geraniol is well-known for its cellular toxicity from antimicrobial activity.^37^ In addition, use of the C_5_ IPP and DMAPP pool for geraniol production may potentially interfere with pigment microalgal metabolism, e.g., pigment biosynthesis. For these reasons, microalgal growth was evaluated to analyze if the presence of geraniol in the culture medium has any inhibiting effect on *C. reinhardtii*. Geraniol-producing lines obtained by expression of *Cr*GES_YFP (F4) were compared in terms of the growth rate with their background UVM4 in autotrophic (HS) or in mixotrophic (TAP) conditions using two light intensities (80 μmol photons m^–2^ s^–1^ low light, LL, and 800 μmol photons m^–2^ s^–1^ high light, HL).

As reported in Figure 5, the accumulation of geraniol does not affect algal growth under any of the tested conditions with similar biomass accumulation between F4 and UVM4 lines. According to the previous literature, a decrease in both carotenoids and chlorophyll concentrations per cell and a decrease in the Chl/Car ratio^38^ were observed in *C. reinhardtii* grown in high light compared to the low-light cultivated ones (Figure 6). However, no significant difference in pigment accumulation was detected in the geraniol-producing line compared to UVM4, excluding negative effects on chlorophyll or carotenoid biosynthesis due to geraniol production. Consistently, the maximum quantum yield of photosystem II (Fv/Fm) was similar between UVM4 and geraniol accumulating line F4: since Fv/Fm measurements are commonly used to reveal stress conditions for the photosynthetic apparatus (Figure 5), it is possible to conclude that heterologous production of geraniol did not cause any negative or inhibitory effects on *C. reinhardtii* cells.

**Figure 5:**
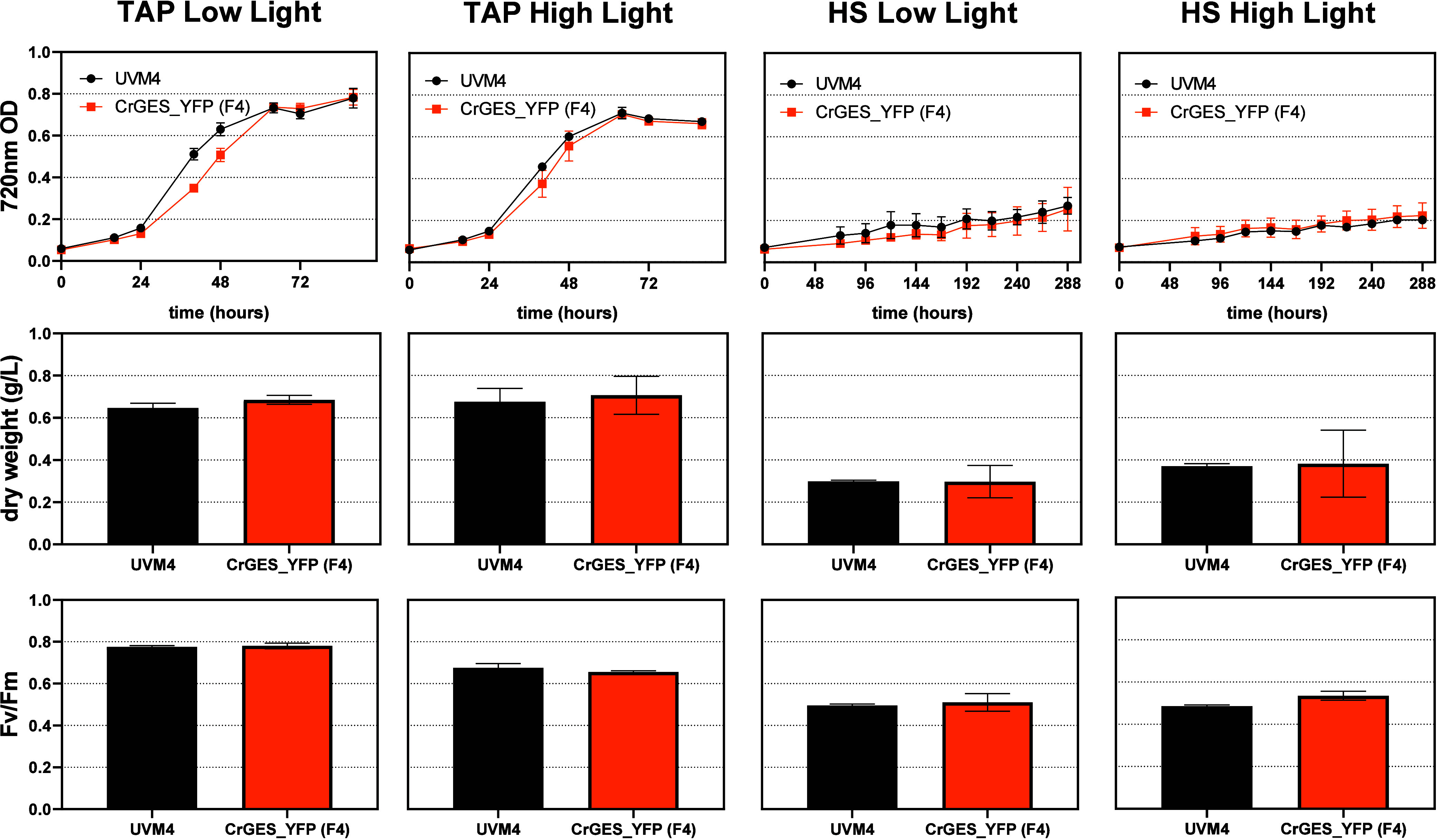
Growth parameters for *Cr*GES_YFP (F4) and UVM4 lines. The growth test was conducted in mixotrophy (TAP) or autotrophy (HS) in low (80 μmol photons m^–2^ s^–1^) or high (800 μmol photons m^–2^ s^–1^) light. 720 nm optical density, dry biomass weight, and Fv/Fm are shown. Values are the average of three independent experiments.

**Figure 6:**
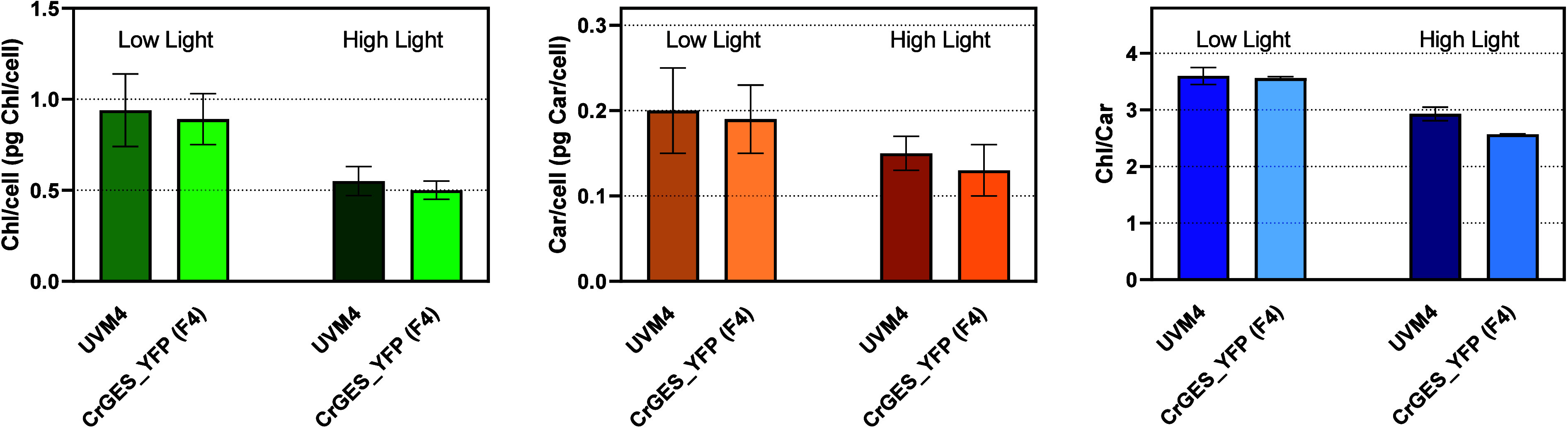
Chlorophyll and carotenoid content of the *Cr*GES_YFP expressing line. Chlorophyll and carotenoid content of the *Cr*GES_YFP expressing line (F4) was compared to the UVM4 case. All parameters were evaluated in low (80 μmol photons m^–2^ s^–1^) or high (500 μmol photons m^–2^ s^–1^) light and reported as an average of three biological replicates. The error bars represent the standard deviation.

Considering that the geraniol titer herein obtained was lower than 100 μg/L, the potential inhibitory effects of higher concentration of geraniol was tested in *C. reinhardtii* cells adding a 1, 10, or 100 mg/L commercial geraniol standard to the growth medium (Supplementary Figure 11). Similar growth curves were obtained in the presence or absence of 1 mg/L geraniol, while partial growth inhibition was evident at a 10 mg/L concentration. Complete inhibition of growth was instead obtained at a 100 mg/L geraniol concentration. The results obtained suggest the possibility to further increase geraniol titer by at least 10-fold compared to the *Cr*GES_YFP expressing lines before inducing growth inhibition.

### Increasing the Amount of GES Enzyme Does Not Result in Geraniol Enhancement

In the case of engineered lines, a linear correlation could be observed between the *Cr*GES protein content and geraniol accumulation (Supplementary Figure 6). A common strategy to increase the catalytic activity of a recombinant enzyme is to insert multiple gene copies into the host genome to increase the total protein amount.^28^ The best geraniol accumulating line (F4) was transformed with a second copy of the *Cr*GES gene, which fuses the enzyme sequence with mCherry. The screening of the *Cr*GES_mCherry lines was performed as in the case of *Cr*GES_YFP lines combining fluorescence and Western blot selecting two expressing *Cr*GES_mCherry lines in a GES_YFP background (Supplementary Figure 12). The localization of both *Cr*GES_YFP and *Cr*GES_mCherry in the chloroplast was confirmed by confocal microscopy (Figure 7). Each integration event underlays individual regulatory mechanisms of expression, resulting in variable protein accumulation. Two *Cr*GES_YFP and *Cr*GES_mCherry expressing lines (D11 and D4) were evaluated to determine the amount of enzyme based on YFP and mCherry fluorescence. As reported in Figure 7, lines expressing two copies of the *Cr*GES enzyme maintain the same YFP signal per cell and thus the same *Cr*GES_YFP accumulation as the F4 background but with an extra amount of enzyme due to *Cr*GES_mCherry. *Cr*GES_YFP and *Cr*GES_mCherry quantification was performed based on calibration curves generated using the isolated fluorescent proteins. Increased *Cr*GES enzyme content could be determined in both D11 and D4 lines compared to the F4 case, with the highest *Cr*GES content in the case of the D11 line. Nevertheless, despite the increase in *Cr*GES enzyme, no increase in geraniol accumulation was reported in either D11 or D4 compared to the *Cr*GES_YFP only expressing line F4 (Figure 7), suggesting a possible shortage in substrate availability or activation of the regulatory mechanism.

**Figure 7:**
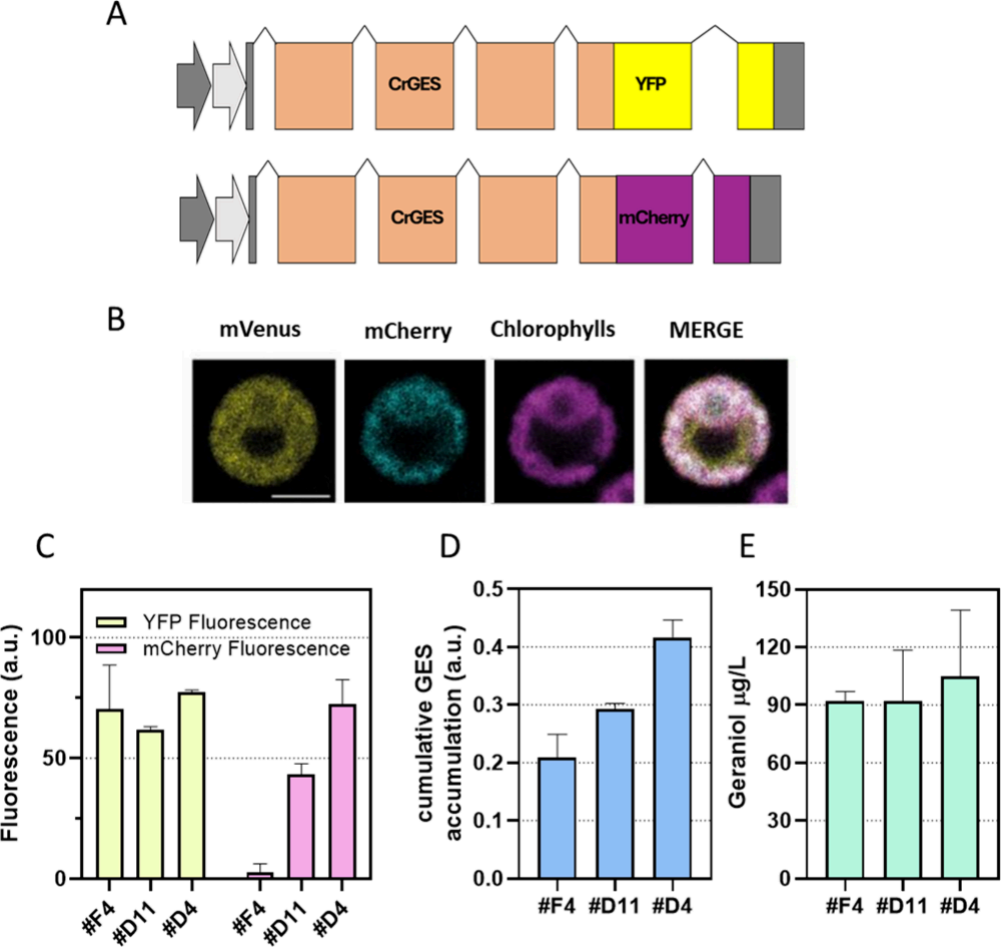
Expression of multiple copies of the GES gene and consequences on geraniol production. (A) Diagrams of expression cassettes used to express two copies of the GES gene. (B) Localization of *Cr*GES_YFP and *Cr*GES_mCherry. YFP fluorescence (mVenus), mCherry, chlorophyll autofluorescence, and the merger of these three channels are shown. Excitation for YFP was 514 nm, 543 nm for mCherry, and 633 nm for chlorophylls. Emission was detected at 522–572 nm for YFP, 560–620 nm for mCherry, and 670–690 nm for chlorophylls. The scale bar represents 5 μm. YFP and mCherry fluorescence (C), *Cr*GES accumulation (D), and geraniol production (E) of the two double-expressing lines in comparison with those of the parental F4 line. *Cr*GES accumulation was deducted by measuring the fluorescence compared with the calibration lines obtained with known amounts of isolated fluorophores.

### Increased GPP Availability Results in Increased Geraniol Accumulation

The C_10_ molecule GPP is the substrate used by GES to produce geraniol. The condensation of C_5_ units to GPP has already been identified as a limiting step in terpenoid production.^39,40^ The prenyltransferase geranyl diphosphate synthase (GPPS) catalyzes the formation of GPP from equimolar quantities of IPP and DMAPP. Our results demonstrated that a GPP pool is present in the plastid but likely limits *Cr*GES activity. To overcome this limitation, the GPP synthase (GPPS) from *Lithospermum erythrorhizon* (*Le*GPPS) was chosen as a candidate for expression: this enzyme was indeed the only one with reported cytosolic localization^41^ and was selected to investigate the presence of a cytosolic IPP/DMAPP pool with the possibility to direct it into the chloroplast if necessary. Gene optimization was performed as previously described for the *Cr*GES gene by combining codon usage optimization, intron insertion, and fluorophore fusion. *Le*GPPS protein sequence does not contain any transit peptides recognized in *C. reinhardtii*, according to PredAlgo, consistently with previous predicted cytosolic localization.^41^ Accordingly, when *Le*GPPS was expressed in the *C. reinhardtii* UVM4 background fused at the C-terminus with YFP, (Figure 8A and Supplementary Figure 13) by confocal microscopy, it was possible to confirm the cytosolic localization of the *Le*GPPS_YFP fused protein (Figure 8B, first line). GPPS was thus expressed in the cytosol or in the chloroplast of *Cr*GES expressing lines as previously described (Supplementary Figures 14 and 15). In the first case, the low YFP fluorescence signal found in the *Cr*GES_YFP expressing line allowed to quickly identify lines coexpressing *Cr*GES and *Le*GPPS measuring YFP fluorescence signal due to simultaneous expression of both *Cr*GES_YFP and *Le*GPPS_YFP. The results obtained were then confirmed by Western blot. The presence of the same fluorophore does not allow us to perform *Le*GPPS_YFP localization by confocal microscopy. However, a precise *Le*GPPS_YFP cytosolic localization was demonstrated in UVM4 and can also be assumed in the case of the chloroplast *Cr*GES expressing a background. On the contrary, in cytosolic coexpression of *Cr*GES and *Le*GPPS, enzymes were fused to YFP and mCherry, respectively. Lines were generated as previously, screened by YFP and mCherry fluorescence and confirmed by Western blot. The presence of distinct fluorophores allows protein localization by confocal microscopy (Supplementary Figure 15). Considering that geraniol was accumulated only when *Cr*GES was localized in the chloroplast, *Le*GPPS fused to mCherry was also targeted into the same compartment using the previously described PSAD transit peptide. *Le*GPPS_mCherry was thus expressed in the best *Cr*GES expressing line (F4) (Supplementary Figure 16), and by confocal microscopy, it was possible to observe a colocalization of *Cr*GES_YFP and *Le*GPPS_mCherry in the chloroplast (Figure 8B).

**Figure 8:**
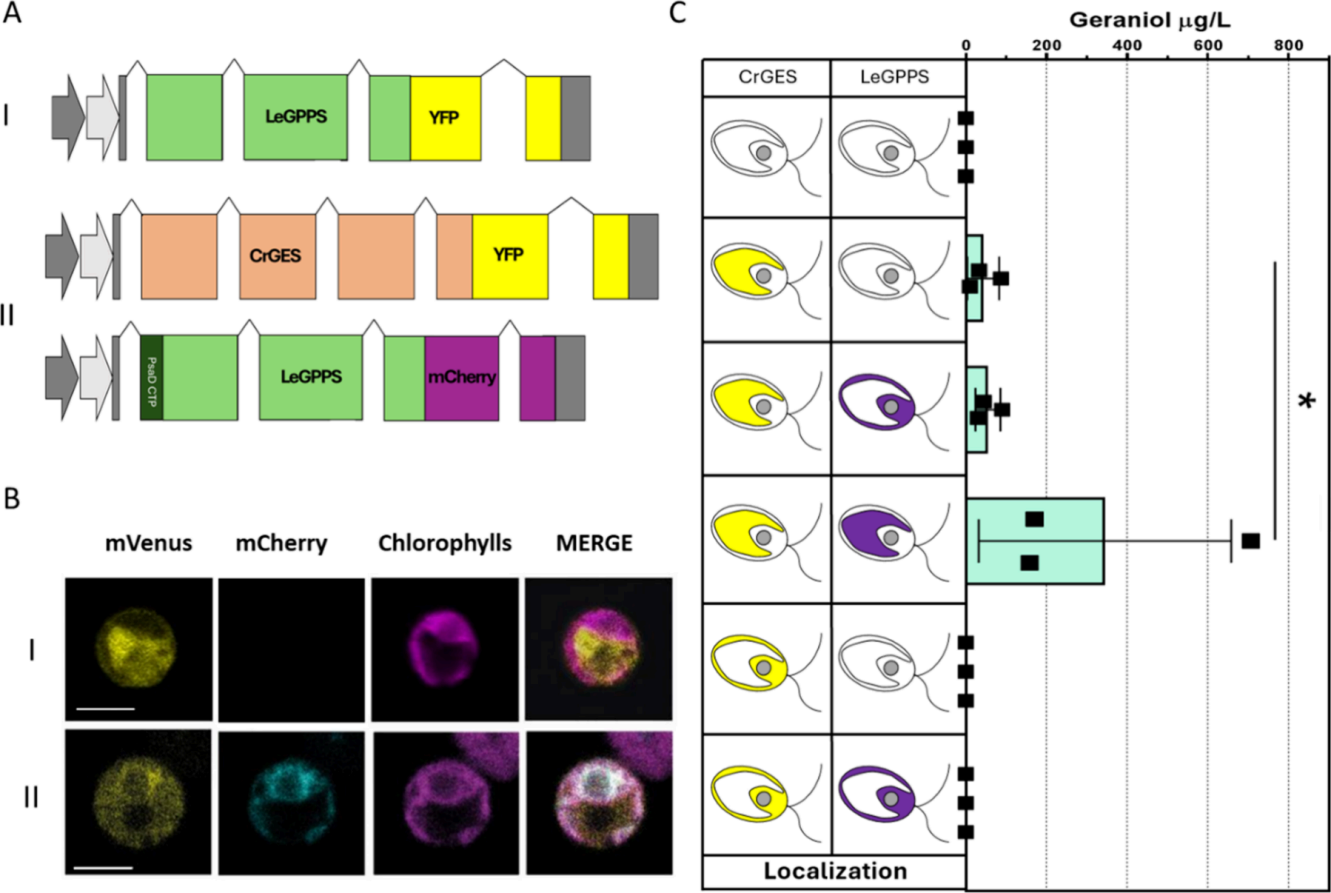
Combined expression of *Cr*GES and *Le*GPPS and consequences on geraniol production. (A) Diagrams of expression cassettes used for the expression of *Le*GPPS (i) for localization and *Le*GPPS and *Cr*GES for chloroplast double expressing line generation (ii). (B) Localization of *Le*GPPS_YFP and *Le*GPPS_mCherry in lines generated using vector (i, ii). YFP fluorescence (mVenus), mCherry, chlorophyll autofluorescence, and the merger of these three channels are shown. Excitation for YFP was 514 nm, 543 nm for mCherry, and 633 nm for chlorophylls. Emission was detected at 522–572 nm for YFP, 560–620 nm for mCherry, and 670–690 nm for chlorophylls. The scale bar represents 5 μm. (C) Geraniol accumulation in *Cr*GES and *Le*GPPS expressing lines in the different localizations and combination. Localization is schematized based on confocal microscopy results. Geraniol accumulation was evaluated in the cultivation medium only. Each square represents the average of three technical replicates for individual lines. The significantly different values (*P* < 0.05) are indicated with an asterisk (*).

The best lines expressing *Le*GPPS, in both the cytosol and chloroplast, were tested for geraniol production and compared with lines accumulating only the *Cr*GES enzyme. Geraniol production could be detected only when *Cr*GES was localized in the chloroplast: despite the presence of both *Cr*GES and *Le*GPPS in the cytosol, no geraniol could be detected on this line. These findings suggest the absence of the DMAPP and IPP pool in the cytosol available for *Le*GPPS to synthesize GPP. Our results demonstrate that cytosolic IPP/DMAPP or GPP pools, if present, are low in abundance or not usable to produce terpene. Differently, in the case of a diatom, geraniol production was achieved upon cytosolic accumulation of the GES enzyme in *P. tricornutum*.^15^ It is worth noting that in diatoms, both MVA and MEP pathways are active. With MVA being located in the cytosol, the availability of IPP/DMAPP pool is likely far higher compared to *C. reinhardtii*, where these C_5_ substrates are only produced by the MEP pathway in the chloroplast.^42^ When *Le*GPPS was expressed in the cytosol and *Cr*GES in the chloroplast, the geraniol production was similar to the *Cr*GES only expressing line F4 (Figure 8C). In contrast, the localization of both *Cr*GES and *Le*GPPS into the plastid (Figure 8C fourth line) resulted in the production of ∼700 μg/L of geraniol, which is 7.7 folds higher than the titer reached in the case of the *Cr*GES only expressing line.

A further possible strategy to increase geraniol production is to exploit the substrate channeling, directly fusing *Cr*GES and *Le*GPPS enzymes. The passing of the GPP intermediary metabolic product from GPP synthase directly to GES synthase could increase the enzymatic activity and, thus, the geraniol yield. The *Cr*GES and *Le*GPPS sequence was fused with YFP and localized into the chloroplast thanks to the *Cr*GES endogenous transit peptide upon transformation of the UVM4 background (Supplementary Figure 17). However, lower geraniol accumulation in *Cr*GES_*Le*GPPS expressing lines was measured compared to the line expressing only *Cr*GES (Supplementary Figure 17). This is probably caused by incorrect folding, wrong spatial distribution, or a low expression level caused by *Cr*GES and *Le*GPPS fusion.

### Geraniol Production Can Be Further Increased by Acting on the MEP Pathway

Early enzymatic steps of the MEP pathway are known bottlenecks for terpene production: The 1-Deoxy-d-xylulose-5-phosphate synthase (DXS) is the main rate-limiting step in many organisms including cyanobacteria, *E. coli*, and plants.^40,43–45^ Recent studies show that increasing DXS expression boosts terpene production by enhancing the carbon flux toward IPP and DMAPP synthesis.^40,46^ To possibly further boost geraniol production in *C. reinhardtii*, DXS from *Salvia pomifera* (*Sp*DXS) was overexpressed in the chloroplast of the above-mentioned lines expressing both *Cr*GES and *Le*GPPS in the chloroplast (*Cr*GES_YFP + PsaD_*Le*GPPS_mCherry), which shows the highest geraniol accumulation (Figure 9A). The chloroplast localization of *Sp*DXS was first tested in UVM4 (Supplementary Figure 18) using the PSAD transit peptide, as reported by confocal microscopy in Figure 9B. Considering the presence of both YFP and mCherry in the chloroplast of lines expressing *Cr*GES and *Le*GPPS, we used a resistance-based approach to obtain cells expressing *Sp*DXS also. The optimized DXS sequence was directly fused to the aadA gene sequence, conferring resistance to spectinomycin.^47^ This strategy was previously used in *C. reinhardtii* ^28,48^ and allows an easy screening after transformation on the selectable medium. The A3 line, expressing both *Cr*GES and *Le*GPPS into the chloroplast and showing the highest level of geraniol accumulation, was used as a background for *Sp*DXS expression. The growth on a selectable medium implies the expression of the spectinomycin resistance and, thus, *Sp*DXS expression. Fourteen lines expressing *Sp*DXS were tested for geraniol production (Supplementary Figure 19): only a few lines showed a geraniol accumulation higher than background. Expressing the entire enzyme set (*Cr*GES + *Le*GPPS + *Sp*DXS) accumulated in the best cases up to 1 mg/L (1.8 mg/g DW) of geraniol, which is 13- and 1.6-folds higher compared to the only *Cr*GES and the *Cr*GES + *Le*GPPS expressing lines, respectively (Figure 9C). As demonstrated for other terpenes, the expression of *Sp*DXS redirecting the carbon flux toward IPP and DMAPP can ensure a more abundant precursor pool and, consequently, higher monoterpene production. These results confirm that the reaction catalyzed by *Sp*DXS is one of the main limiting steps of the MEP pathway.^40,43–45^

**Figure 9:**
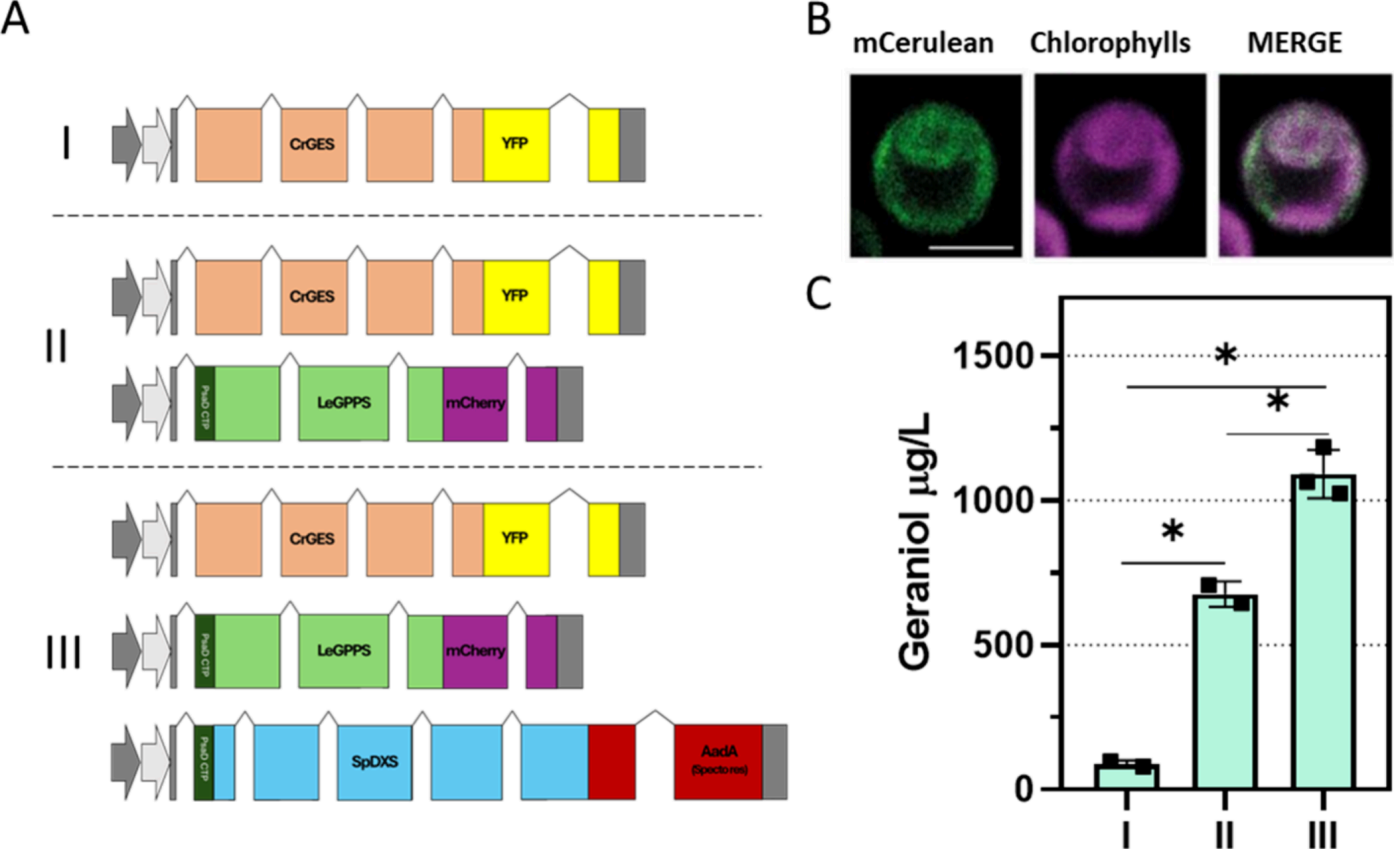
Combined expression of *Cr*GES, *Le*GPPS, and *Sp*DXS and its consequences on geraniol production. (A) Diagrams of expression cassettes used to generate strains expressing *Cr*GES, *Le*GPPS, and *Sp*DXS in panel (C). (B) Localization of *Sp*DXS_CFP in a line appositely generated by using the *Sp*DXS sequence fused to CFP. CFP (mCerulean), chlorophyll autofluorescence, and the merger of these two channels are shown. Excitation for mCerulean was 405 nm and 633 nm for chlorophylls. Emission was detected at 470–540 nm for mCeruean and 670–690 nm for chlorophylls. The scale bar represents 5 μm. (C) Geraniol accumulation in *Cr*GES, *Le*GPPS, and *Sp*DXS expressing lines generated using the vectors in panel (A). The best line generated with the vector in (I) was used as background introducing the *Le*GPPS (II), and the best of these was selected for the insertion of the third enzyme (III). The best line for (I) and (II) enzymatic configuration was reported in the graph, while for (III), the best three were reported. Geraniol accumulation was evaluated in the cultivation medium only. Each square represents the average of two technical replicates for individual lines in the case of (III) and individual analysis for the same genotype for (I) and (II). The significantly different values (*P* < 0.05) are indicated with an asterisk (*).

Considering the far higher geraniol accumulation compared to the single *Cr*GES expressing lines, the possible influence of geraniol on algal growth was also tested in the case of the *Cr*GES + *Le*GPPS + *Sp*DXS expressing lines. Growth parameters, as well as pigment content, were evaluated in the G7 best geraniol accumulating line. Growth tests were performed in both autotrophy (HS) and mixotrophy (TAP) under low light (80 μmol photonsm^–2^s^–1^) and high light (800 μmol photons m^–2^s^–1^). As in the case of the single *Cr*GES expressing line F4, also in the G7 line, accumulating both *Cr*GES, *Le*GPPS, and *Sp*DXS, the same growth curve, biomass accumulation and pigment content were observed compared to the background UVM4 (Supplementary Figures 20 and 21). No effects on *C. reinhardtii* algal growth were observed in all tested conditions, consistently with the results obtained with 1 mg/L of geraniol externally added to the growth medium (Supplementary Figure 11).

### Dodecane Is Not Efficient in Extracting Geraniol from the Microalgal Culture

Geraniol produced by engineered *C. reinhardtii* cells was mainly released into the growth medium, but 48 h after inoculation, its presence in the aqueous phase dramatically decreased. Similar behavior was observed 34 h after inoculation in *E. coli* strains engineered to produce geraniol.^31^ Chacón and co-workers hypothesized that endogenous *E. coli* enzymes convert geraniol into nerol and citronellol. However, at the same time, they showed an ∼30% reduction in geraniol content in a solvent-free system without microorganism cultivation and thus due to evaporation. The same result was obtained by Liu and a co-worker, showing a decrease in geraniol concentration due to evaporation.^11^ Usually, microbial terpenes can be extracted using a hydrophobic biocompatible solvent. To overcome the loss of geraniol due to evaporation, an aqueous–organic two-phase system with dodecane was tested.^31,49^ Two-phase cultivation allows constant extraction from living cells, with hydrophobic solvents acting as a sink for hydrophobic molecules. Decane, dodecane, isopropyl myristate, and in recent years also perfluorinated compounds^50^ are commonly used for the separation of products from culture, alleviating toxic effects or product inhibition and providing an easy way to collect volatile molecules. Here, dodecane two-phase cultivation was tested to extract geraniol from the microalgal culture as previously done for the same metabolite in other hosts.^31,49^ Dodecane was tested on lines expressing *Cr*GES (F4), *Cr*GES + *Le*GPPS (A3), or *Cr*GES + *Le*GPPS + *Sp*DXS (G7) 48 h after inoculation, in which the maximum accumulation in the supernatant was previously recorded. As reported in Figure 10A, the solvent overlay on top of the algal culture was able to capture the geraniol present in the supernatant, reaching the maximum concentration of 1.1 mg/L culture in the case of the line overexpressing *Cr*GES, *Le*GPPS, and *Sp*DXS. Consistently with the production data obtained in the absence of dodecane (Figure 9C), the *Cr*GES + *Le*GPPS + *Sp*DXS expressing line yielded the highest geraniol titer in the dodecane phase. The capturing ability of dodecane was then tested by using a geraniol standard solution (Figure 10B,C). Geraniol (50 μg) was added into flasks containing the two-phase TAP-dodecane in the presence or absence of UVM4 algal cells. Surprisingly, the dodecane’s ability to trap the geraniol was limited in our conditions: ∼70% of geraniol left the aqueous phase after 12 h, but only 3.5% of it (1.1 ± 0.064 μg) was trapped by the dodecane phase. After 72 h, the amount of geraniol in the water phase was reduced by a further 5% with no concentration increase in the solvent, suggesting evaporation from the dodecane layer (Figure 10B). Interestingly, the percentage of geraniol that left the water phase is higher when using the two-phase system: ∼70% in the presence of dodecane with respect to ∼30% in the absence after 12 h (Supplementary Figure 4 and Figure 10B). This can result from favored evaporation due to the presence of dodecane, which acts as a cosolvent. The same experiment was conducted in the presence of UVM4 algal cells (Figure 10C); again, 50 μg of commercial geraniol was added to the culture medium, and both TAP and dodecane phases were sampled after 12 and 72 h. ∼80% of geraniol left the water phase after 12 h of cultivation, but in this case, the dodecane layer was able to trap only 0.75% of the added amount (0.3 ± 0.025 μg of 39.6 ± 0.9 μg). After 72 h, the geraniol in the water phase was reduced by ∼98% with respect to the initial value with the dodecane layer being able to trap only 0.06% (0.03 ± 0.017 μg). The results obtained demonstrate that the geraniol-decreased concentration is due to degradation by microalgal cells and evaporation from the growth medium. It can be hypothesized that *C. reinhardtii* cells have a faster geraniol degradation rate compared with the capacity of dodecane to extract this terpene from the aqueous phase. The use of other solvents with a higher geraniol affinity could be tested, even if the use of more hydrophobic solvents could partially inhibit microalgal growth. However, despite being inefficient in capturing geraniol, the two-phase dodecane system herein tested gives an indication on actual geraniol productivities. Considering that less than 3.5% of the geraniol transferred from the water phase to the dodecane layer is efficiently trapped by the solvent (Figure 10B), it is possible to hypothesize that the actual amount of geraniol produced by transformed microalgae and transferred to the dodecane layer could be in the range of tens of mg/L of culture. So far, it remains speculation to be further tested by additional research effort.

**Figure 10:**
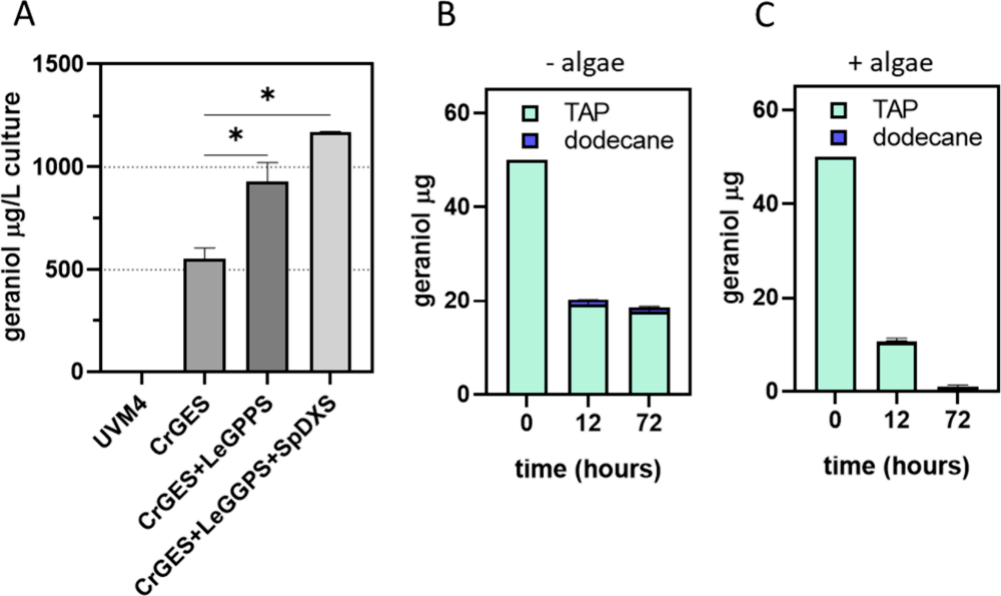
Geraniol accumulation in an aqueous–organic two-phase system. (A) Accumulation in the 5% dodecane overlay referred to cultivation volume. Geraniol capturing by dodecane overlay in the absence of microalgae (B) or in the presence (C). The significantly different values (*P* < 0.05) are indicated with an asterisk (*).

## Conclusions

The search for alternative methods to produce geraniol is a much-discussed topic.^11–15,34,51,52^ So far, different genera and species of microorganisms have been studied as hosts for bioproduction. For example, in *S. cerevisiae*, through metabolic engineering, researchers yielded 1.68 g/L of geraniol by fed-batch cultivation while using *E. coli* as a fermentation chassis, researchers reached a concentration of 13.19 g/L of geraniol, which currently appears to be the highest concentration reported in the literature.^14^ Attempts to use photoautotrophic microorganisms such as microalgae, considering their ability to fix CO_2_ and their good carbon flux directed toward the production of terpenes, have recently been reported in *P. tricornutum* able to accumulate 0.3 mg/L of geraniol.^15^ Photosynthetic production of other monoterpenes, such as limonene and β-phellandrene, but not geraniol to our knowledge, was successfully achieved in cyanobacteria, where rational metabolic engineering led to an accumulation of 4 mg/L for the former and 24 mg/g (of dry biomass) for the latter.^53–59^ *C. reinhardtii* allows the exploitation of several biotechnological tools available for metabolic engineering to boost gene expression and redirect metabolic flux toward the production of interest.^17,19,60,61^ Here, *C. reinhardtii* cells transformed with the complete recombinant enzymatic set *Cr*GES, *Le*GPPS, and *Sp*DXS expressed in the chloroplast could accumulate up to 1 mg/L of geraniol. To the best of our knowledge, this is the highest production obtained in a microbial photosynthetic host, 3.6-fold higher than previous metabolic engineering approaches reported in *P. tricornutum*. It is noteworthy that partial inhibition of algal growth was observed at 10 mg/L, thus far higher concentration compared to the maximum 1 mg/L production titer herein obtained. This result suggests potential additional possibilities to increase geraniol titer without impairing *C. reinhardtii* growth. However, much higher geraniol production can be obtained by heterotrophic growth of engineered yeasts or bacteria.^11–14^

Nevertheless, we demonstrate that a considerable part of the geraniol produced is lost by evaporation or used by microalgae, making it impossible to precisely determine the actual productivity of our system. *C. reinhardtii* can successfully produce monoterpenes; however, efforts should focus on optimizing their capture. This can be achieved by leveraging specific compounds, such as the recently reported fluorocarbons, which, due to their chemical structure, preferentially capture monoterpenes over higher molecular weight terpenes.^50^ The results described in this work are still an encouraging starting point for effective geraniol production in a green, sustainable system. Circular economy and sustainability are some of the most important pillars of modern society; therefore, finding new alternatives to the synthesis of products of interest without harming our environment is undoubtedly one of the main issues for the coming years. A further application of the results herein reported is related to the food sector: geraniol accumulation in tomatoes was reported to improve the flavor of the fruits;^62^ geraniol accumulation in microalgae could thus be a possible strategy to improve the taste of algal biomass and algae-derived products, which is one of the limiting factors for algae application in the food industry.

## Materials and Methods

### *C. reinhardtii* Cultivation

Cultivation of *C. reinhardtii* was conducted in Tris-acetate-phosphate (TAP) or high-salt (HS) minimal media.^63^ Isolated expressing lines were maintained on TAP agar plates under a continuous white LED light set at 40 μmol photons m^–2^ s^–1^. Growth in the liquid TAP medium for geraniol production was conducted in flasks under 160 rpm agitation at 23 °C in continuous white light (90 μmol of photons m^–2^ s^–1^). Growth test conditions to evaluate the performance of expressing strains with respect to UVM4 are indicated in the main text or in the dedicated section. The cell density was measured using Countess 3 Automated Cell Counter (Thermo Scientific, USA).

### Construct Design, Cloning Steps, Transformation, and Colony Screening

*C. reinhardtii* UVM4 ^64^ was used as a background strain for nuclear engineering. Heterologous protein sequences expressed in *C. reinhardtii* UVM4 are reported in Supplementary Figure 22. The CDS of *Cr*GES (Uniprot: J9PZR5), *Le*GPPS (BBG62184.1), and *Sp*DXS (A0A4P8L6B4) were codon-optimized for the host species, and the sequences of RBCS2 intron 1 were inserted to enhance the expression in *Chlamydomonas*.^19^ Synthetic genes were synthesized through the GeneArt service of Thermo Scientific (USA). All cloning steps were performed with Thermo FastDigest restriction enzymes, followed by ligation into pOpt2 vectors.^16,26^ All expression cassettes used in this work are summarized in Supplementary Table 1. Plasmid sequences and codon-optimized sequences for the synthetic genes used for *C. reinhardtii* transformation are reported in Supplementary File 1. Plasmid maps are reported in Supplementary File 2. For transformation, 10 μg of vector DNA was linearized with XbaI and *Kpn*I restriction enzymes prior to transformation of *C. reinhardtii* using a glass bead method.^65^ Transformants were selected using TAP agar plates supplied with respective antibiotics (12 mg/L paromomycin, 35 mg/L hygromycin, and 200 mg/L spectinomycin). Antibiotic-resistant colonies were prescreened by fluorescence, and the most fluorescent lines were then individually cultured and analyzed by SDS-PAGE, followed by Western blotting and immunodetection. Positive lines showing a band at the expected molecular weight were used for further analysis.

### Fluorescence Microscopy Localization

YFP, mCherry, and CFP fluorescence subcellular imaging were performed by confocal microscopy as previously reported.^48,66^ Images were recorded using a Leica TCS-SP5 inverted confocal microscope (Leica Mycrosystems, Germany). Excitation was performed at 524, 543, and 405 nm, while detection was performed at 522–572, 560–620, and 470–540 nm for YFP, mCherry, and mCerulean, respectively. Chlorophylls were exited at 524 nm, and their fluorescence was detected at 680–720 nm.

### Growth Analysis

Growth in shaking flasks was assessed by daily monitoring of the OD_720 nm_. 5 × 10^5^ cells/m measured using Countess 3 Automated Cell Counter (Thermo Scientific, USA)­l were inoculated in 50 mL of TAP and HS medium and were cultivated under both low light (80 μmol photons m^–2^ s^–1^) and high light (800 μmol photons m^–2^ s^–1^) at 160 rpm agitation until the stationary phase was reached. The cellular dry weight was measured by gravimetric analysis on an oven-dried culture. The quantum yield of photosystem II (Fv/Fm) was calculated by using dual-PAM-100 (Walz, Germany) on dark-adapted cells.

### Absorption Spectra and Quantification of Pigments

Pigments were extracted from biomass pellets by resuspension in 80% acetone followed by analysis of absorption spectrum with a Jasco V-550 UV/vis spectrophotometer followed by curve fitting as previously described.^67^

### Enzyme Accumulation Quantification

Protein accumulation comparisons were carried out based on fluorescence, considering the presence of enzymes of interest as a fusion protein with fluorophores. In the case of two different fluorophores simultaneously present, calibration curves were generated using the isolated fluorescent proteins, and the enzyme quantification was calculated based on them.

### Geraniol Quantification

Geraniol-producing strains were cultivated in shake flasks in the TAP medium at 160 rpm and 25 °C and 800 μmol photons m^–2^s^–1^. Cells were separated from the supernatant, and both were analyzed using GC-MS. Intracellular quantification was performed by adding 100 μL of internal standard 2-octanol (4.2 mg/L in ethanol) and extracting twice with 1.5 mL of CH_2_Cl_2_. The fractions were then combined and concentrated under a gentle nitrogen stream, to reach 500 μL. One microliter extract was then injected in splitless mode into a GC-MS system HP 7890A (Agilent Technologies) gas chromatograph coupled to a 5977B quadrupole mass spectrometer, equipped with a Gerstel MPS3 auto sampler (Müllheim/Ruhr, Germany). Separation was performed using a DB-WAX UI capillary column (30 m _ 0.25, 0.25 _m film thickness, Agilent Technologies) and helium (6.0 grade) as carrier gas at 1.2 mL/min of constant flow rate. The oven temperature was initially set at 40 °C for 3 min, raised to 230 °C at 4 °C/min, and kept for 20 min. Ionization (EI) was performed at 70 eV with an ion source and quadrupole temperature set at 250 and 150 °C, respectively. The acquisition mode was synchronous SCAN (*m*/*z* 40–200) and single ion monitoring (SIM).

Moreover, extracellular quantification was performed by SPME-GC-MS analysis as described by Slaghenaufi et al.^68^

### Dodecane Geraniol Capture and Analysis

Microalgae were cultivated in the TAP medium at 160 rpm 25 °C and 800 μmol photons/m^2^s with a 5% v/v dodecane overlay as previously reported.^21^ Dodecane raw fraction was purified by pipetting and centrifugation. Clean dodecane samples were then analyzed by GC-MS as described below to assess the amount of geraniol captured.

Solvent samples were analyzed using an Agilent 7890A gas chromatograph (GC) equipped with a DB-5MS column (Agilent J&W, USA) attached to a 5975C mass spectrometer (MS) with a triple-axis detector (Agilent Technologies, USA). A previously described GC oven temperature protocol was used.^50^ After a 13 min solvent delay, mass spectra were first recorded in scan mode in the range 50–550 *m*/*z* at 20 scans s^–1^ and subsequently in selected ion monitoring (SIM) mode (59, 63, and 92 *m*/*z*). In both modes, a geraniol standard calibration curve in the range of 15–500 μg/mL geraniol in dodecane was used for quantification. Chromatograms were processed and integrated using MassHunter Workstation software v. B.08.00 (Agilent Technologies, USA), and geraniol peaks in scan mode were identified by comparing their mass spectra against those of the National Institute of Standards and Technology (NIST) library (Gaithersburg, USA). Chromatograms and peak area integrations were manually inspected for quality control. All GC-MS measurements were performed in technical duplicates (*n* = 2).

### Statistics Analysis

Statistical analysis was performed by using a two-sided Student’s *t* test or one-way ANOVA with posthoc Tukey test in the case of multiple comparisons

## Supplementary Material







## Notes

Features of the different constructs (Supplementary Table 1); data set features (Supplementary Figures 1–22); various experimental details, such as, the screening of different gene-expressing lines (Supplementary Figures 1, 7–10, and 12–18), GC/MS peak identification (Supplementary Figure 2), *Cr*GES expression over time (Supplementary Figure 3), analyses of volatilization of geraniol externally provided (Supplementary Figure 4), accumulation of different monoterpenes in *Cr*GES expressing lines (Supplementary Figure 5), and correlations between *Cr*GES gene expression and geraniol production levels (Supplementary Figure 6); toxicity of geraniol on algal cell growth (Supplementary Figure 11) and performance of coexpression and triple-expression lines (expressing *Cr*GES, *Le*GPPS and *Sp*DXS) in terms of geraniol accumulation, growth, and pigment profiles (Supplementary Figures 18–20); and (Supplementary Figure 22) protein sequences of overexpressed enzymes (PDF)
Plasmids, genes, and codon-optimized sequences for the synthetic genes used in the transformation of *C. reinhardtii* (TXT)
Plasmid maps (PDF)
