# Supplementary material for: Sustainable Production of Bio-Based Geraniol: Heterologous Expression of Early Terpenoid Pathway Enzymes in Chlamydomonas reinhardtii
Source: ACS Synth Biol. 2025 Aug 26;14(9):3753–66. doi: 10.1021/acssynbio.5c00510 (PMC12455652; doi:10.1021/acssynbio.5c00510)
Supplement: Supplementary file 1 [file sb5c00510_si_001.pdf]

Sustainable production of bio-based geraniol: heterologous expression of early terpenoid pathway enzymes in *Chlamydomonas reinhardtii*

Federico Perozeni[1#], Edoardo Ceschi[1#], Giovanni Luzzini[1], Davide Slaghenaufi[1], Matteo Pivato[1], Stefano Cazzaniga[1], Thomas Baier[2], Alexander Einhaus[2], Sebastian Overmans[3], Kyle J. Lauersen[3], Maurizio Ugliano[1], Matteo Ballottari[1*]

[1] *Department of Biotechnology, University of Verona, Strada le Grazie 15, 37134 Verona, Italy*

[2] *Bielefeld University, Faculty of Biology, Center for Biotechnology (CeBiTec), Universitätsstrasse 27, 33615, Bielefeld, Germany.*

[3] *Bioengineering Program, Biological Environmental Sciences and Engineering Division (BESE), King Abdullah University of Science and Technology (KAUST), 239555, Thuwal, Saudi Arabia.*

[‡]These authors contributed equally.

[*]**Corresponding author:** Matteo Ballottari

Email: matteo.ballottari@univr.it

Phone: +39 045 8027823

Supplementary materials

Supplementary Figure 1. *Cr*GES expressing lines screening. Fluorescence screening of *Cr*GES_YFP expressing lines and western blot confirming GES presence in pre-screened lines (insert). Each graph refers to an independent transformation event. Lines are decreasingly ordered with a 40-line gap. For western blot total proteins of 1x10^{6} cells were loaded.

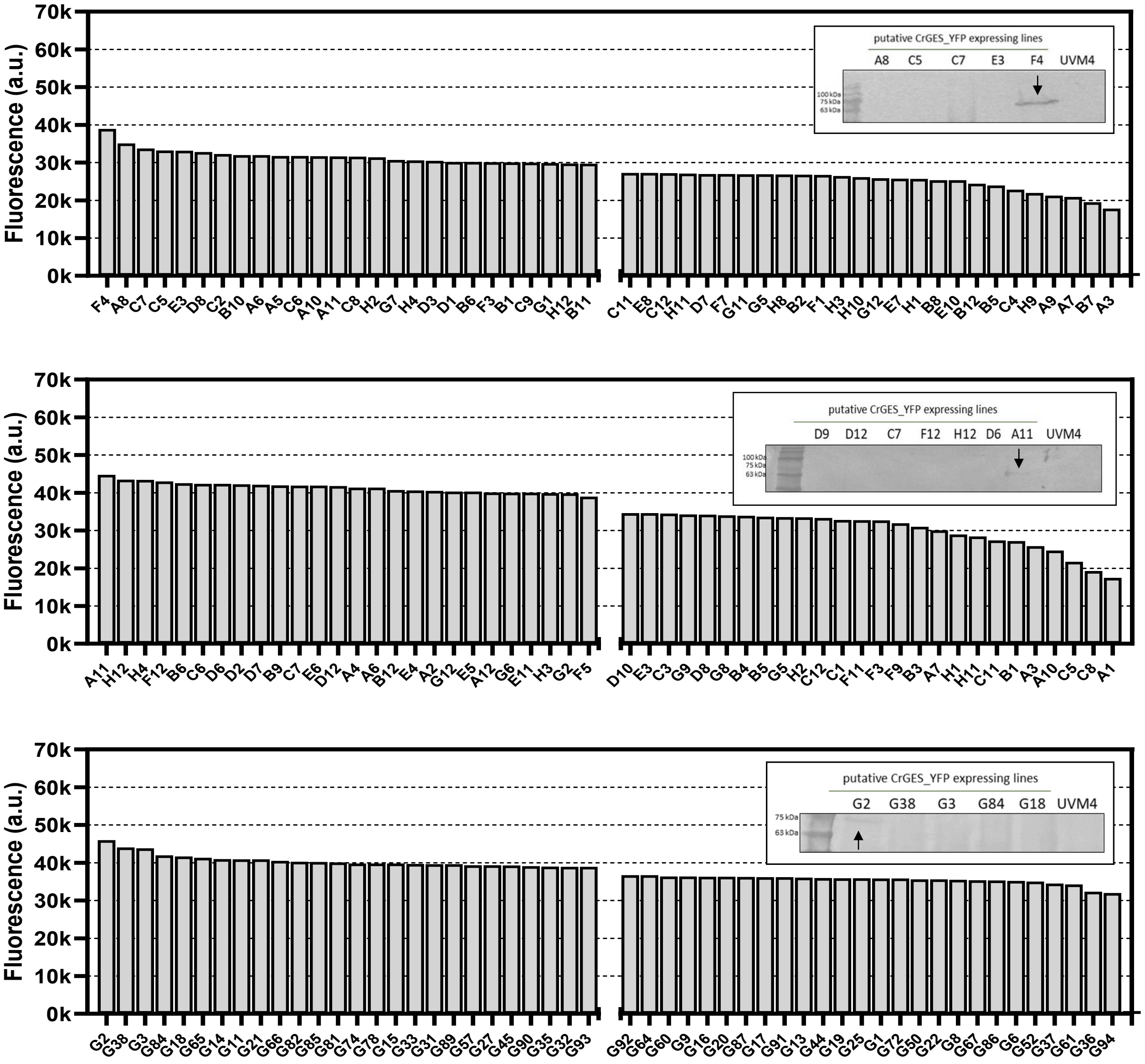

Supplementary Figure 2. Mass fraction of peaks identified by GC/MS in *Chlamydomonas* growth medium.

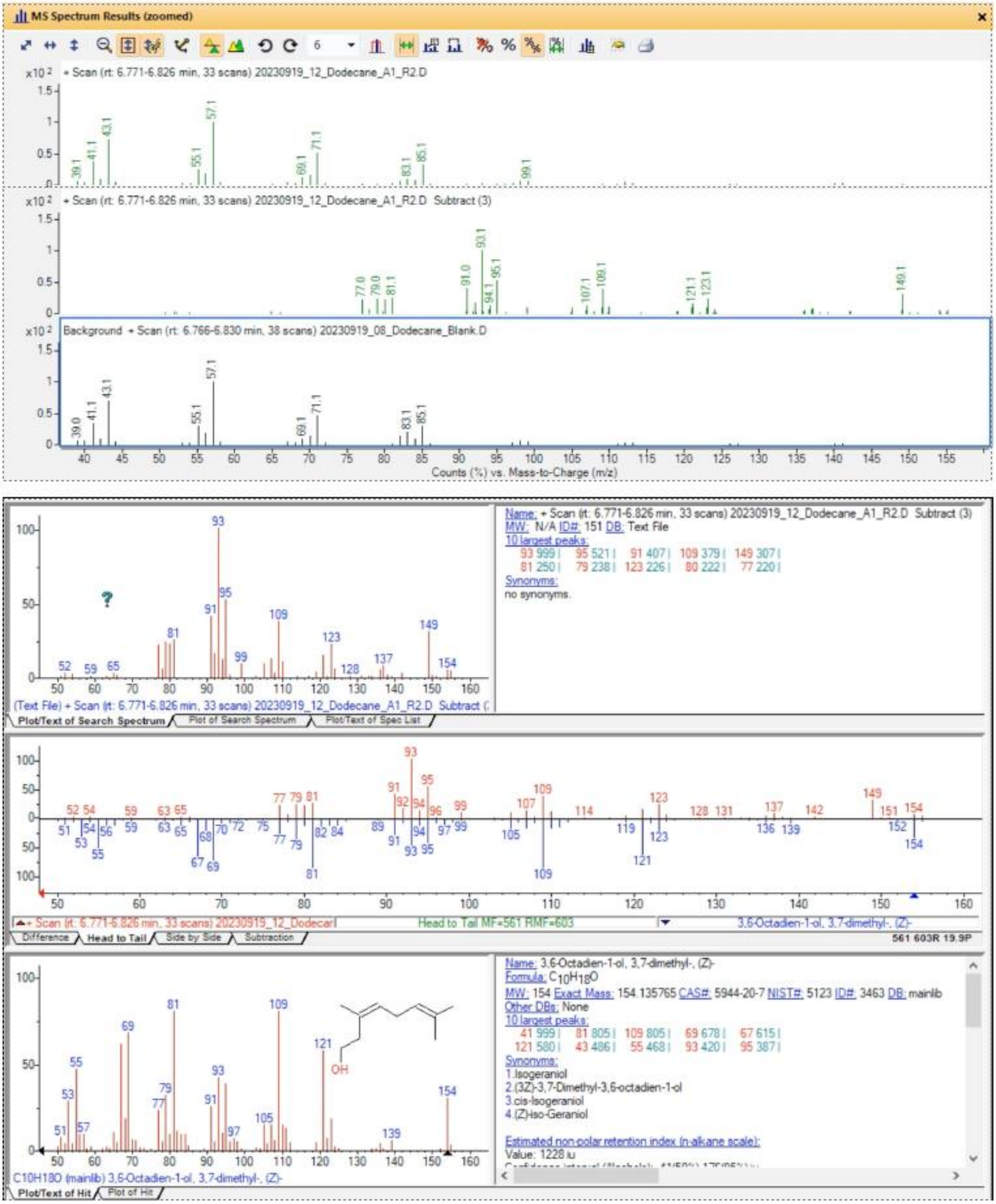

Supplementary Figure 3. *Cr*GES expression during time in *Chlamydomonas* engineered cells. *Cr*GES accumulation per cell based on YFP fluorescence (right axis) and growth curve (left axis).

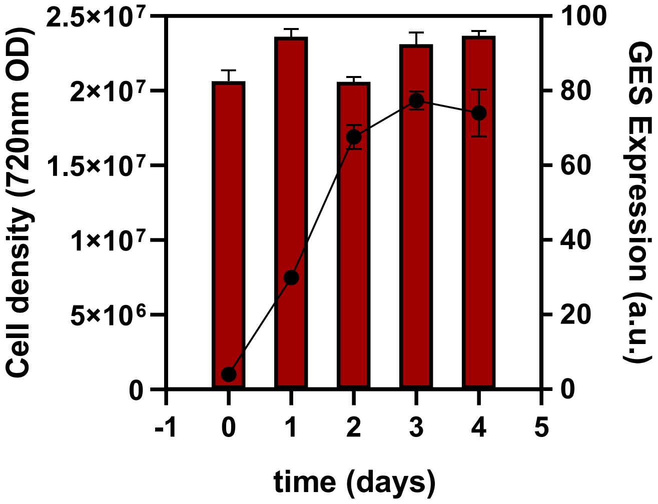

Supplementary Figure 4. Volatilization test. 50µg of geraniol was used as a standard in the absence (left) or presence (right) of UVM4 cells. GC-MS analysis was used to quantify the geraniol abundance in the growth medium. Growth was conducted in mixotrophy using 90 µmol photons/m2s continuous light.

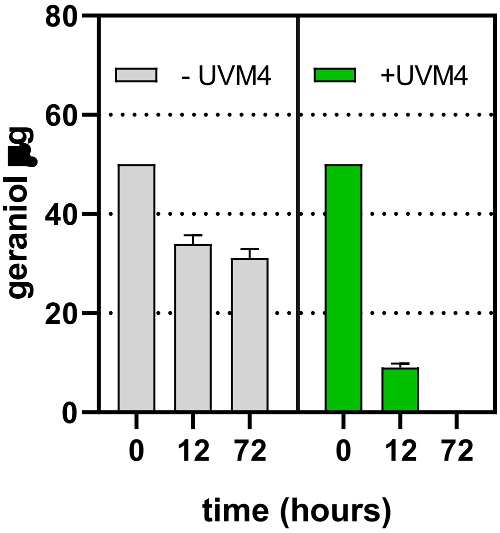

Supplementary Figure 5. Monoterpene accumulation. F4 line was investigated by GC-MS to identify the accumulation of several monoterpene. Growth was conducted in mixotrophy using 90 µmol photons/m2s continuous light.

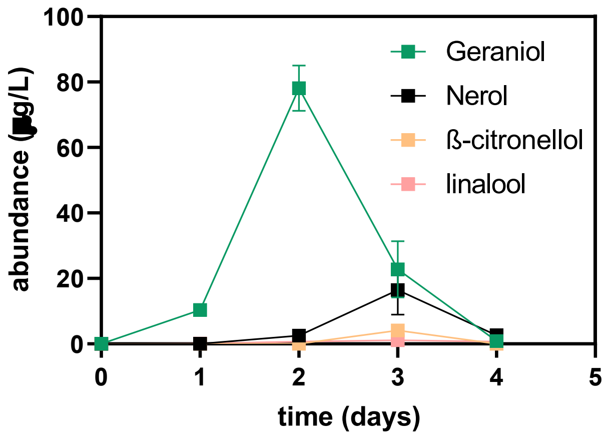

Supplementary Figure 6. Production vs GES expression. Relative geraniol productivity correlated with the fluorescence expression per cell. Colony F4 is indicated. Error bars represent standard deviation.

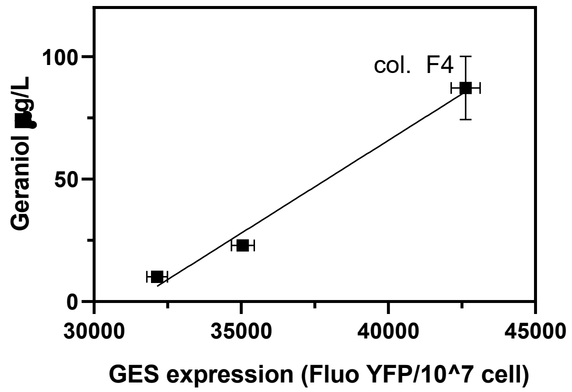

Supplementary Figure 7. PsaD_CrGES expressing lines screening. Fluorescence screening of PsaD_CrGES_YFP expressing lines and western blot confirming GES presence in pre-screened lines (insert). Each graph refers to an independent transformation event. Lines are decreasingly ordered with a 40-line gap if necessary. For western blot total proteins of $1x10^6$ cells were loaded.

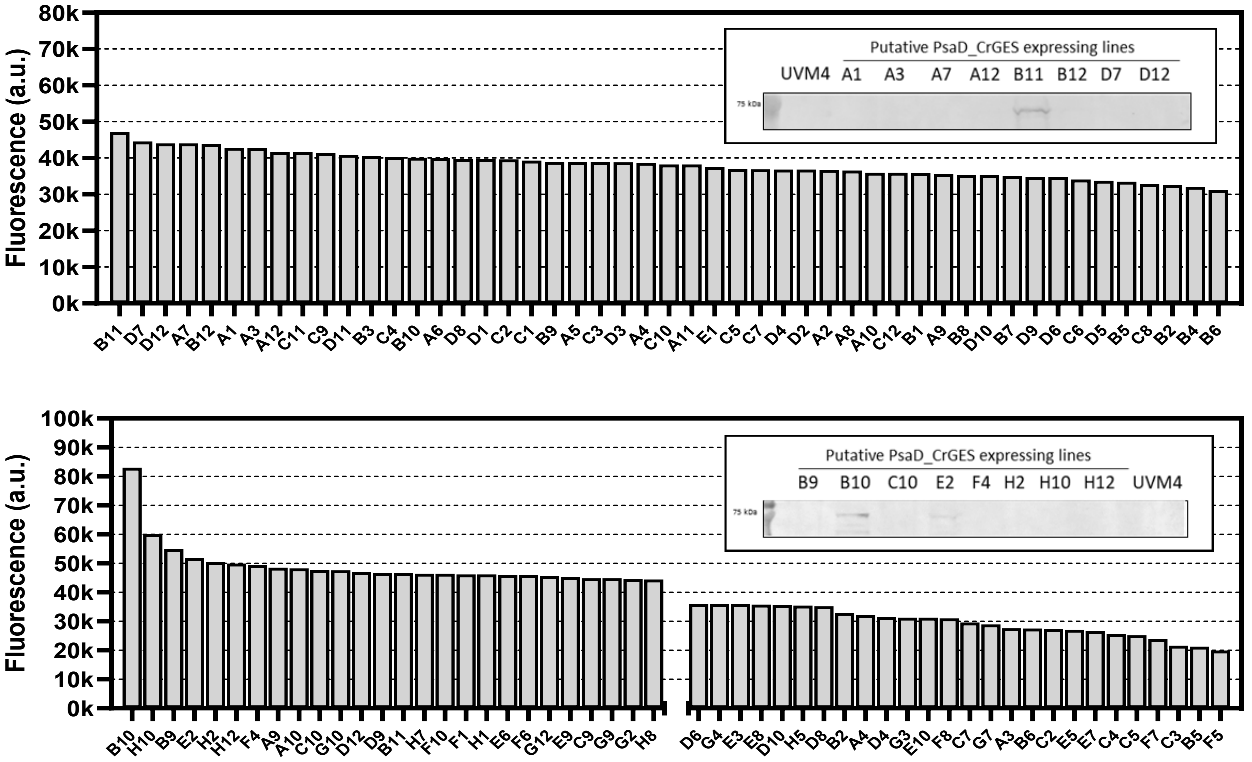

Supplementary Figure 8. PasD_CrGES-43aa expressing lines screening. Fluorescence screening of PsaD_CrGES-43aa_YFP expressing lines and western blot confirming GES presence in pre-screened lines (insert). Lines are decreasingly ordered with a 40-line gap. For western blot total proteins of 1x10^6 cells were loaded.

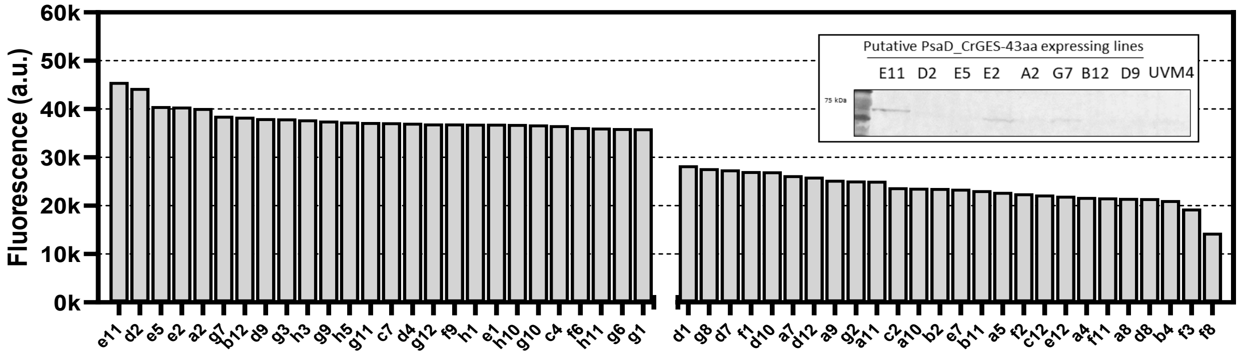

Supplementary Figure 9. *Cr*GES-43aa expressing lines screening. Fluorescence screening of *Cr*GES-43aa_YFP expressing lines and western blot confirming GES presence in pre-screened lines (insert). Lines are decreasingly ordered with a 40-line gap. For western blot total proteins of 1x10^6 cells were loaded.

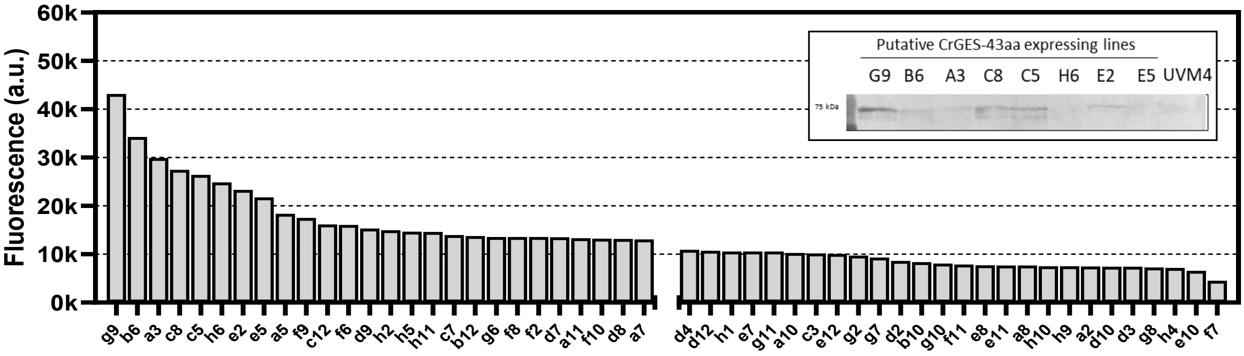

Supplementary Figure 10. GES transit peptide expressing lines screening. (a) Fluorescence screening of putative lines expressing YFP targeted with 43, 60, and 100 N-terminal amino acids of GES protein. Lines are decreasingly ordered with a 40-line gap if necessary. (b) Western blot confirming the presence of the protein in pre-screened lines. Total proteins of 1x10^6 cells were loaded.

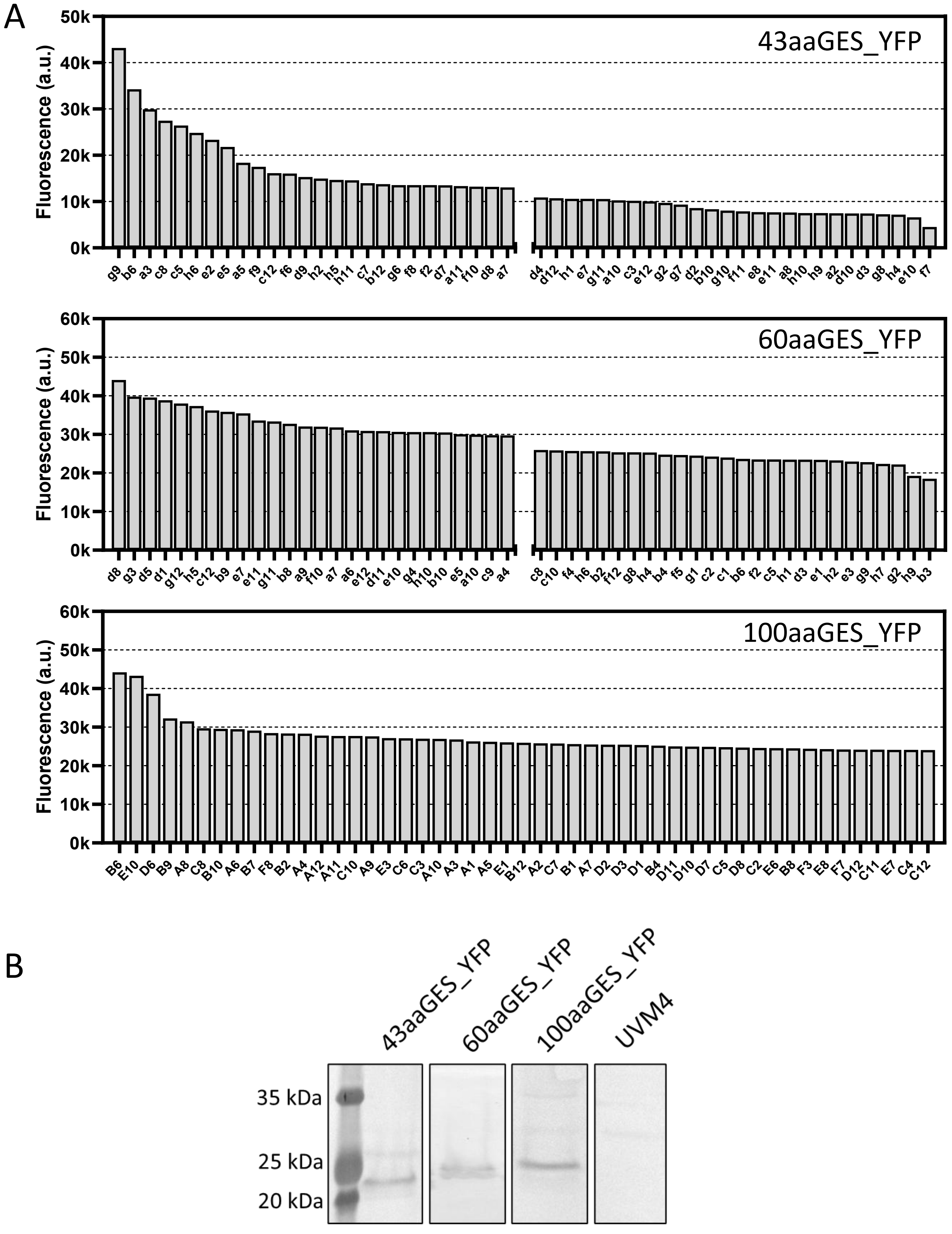

Supplementary Figure 11. Geraniol toxicity evaluation. Growth test was conducted in mixotrophy (TAP) in low (80 µmol photons m^{-2} s^{-1}). 1, 10 or 100 mg/L of geraniol standard was added (A). As control, same amount of methanol was used. Cell density was daily evaluated to recontract growth curve. Representative culture flasks with tested geraniol concentration after 4 days of cultivation (B).

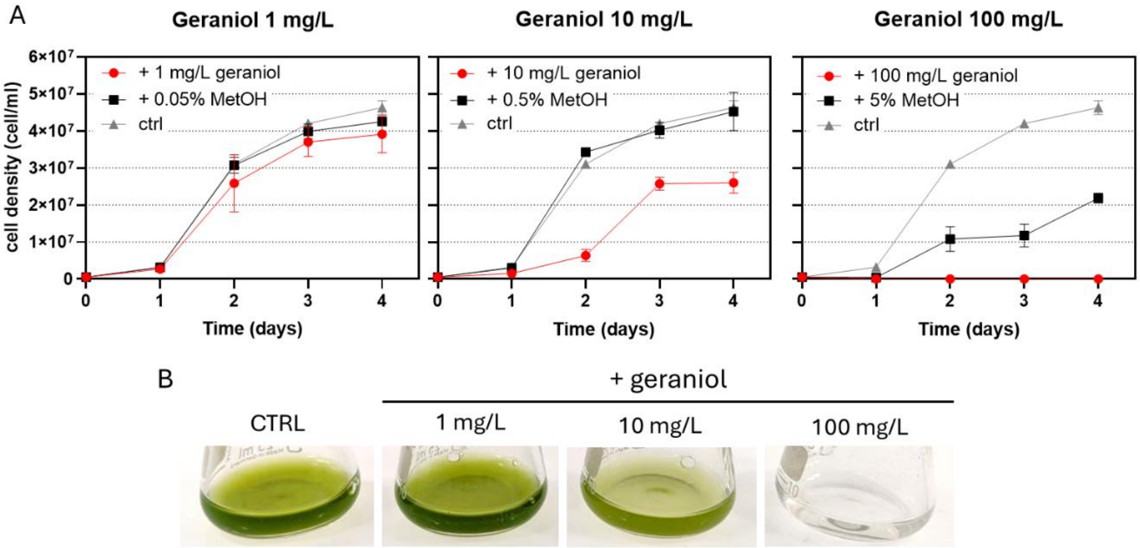

Supplementary Figure 12. *Cr*GES+ *Cr*GES expressing lines screening. Fluorescence screening of putative lines expressing two copies of *Cr*GES. YFP signal on top, mCherry in the middle, and mCherry/YFP ratio on bottom. Lines are decreasingly ordered with a 40-line. Western blot confirming the presence of both proteins in the best two pre-screened lines is reported in the insert (anti-YFP on top and anti-mCherry in the middle). Total proteins of 1x10^{6} cells were loaded. F4 background, best GES expressing lines, and UVM4 are loaded.

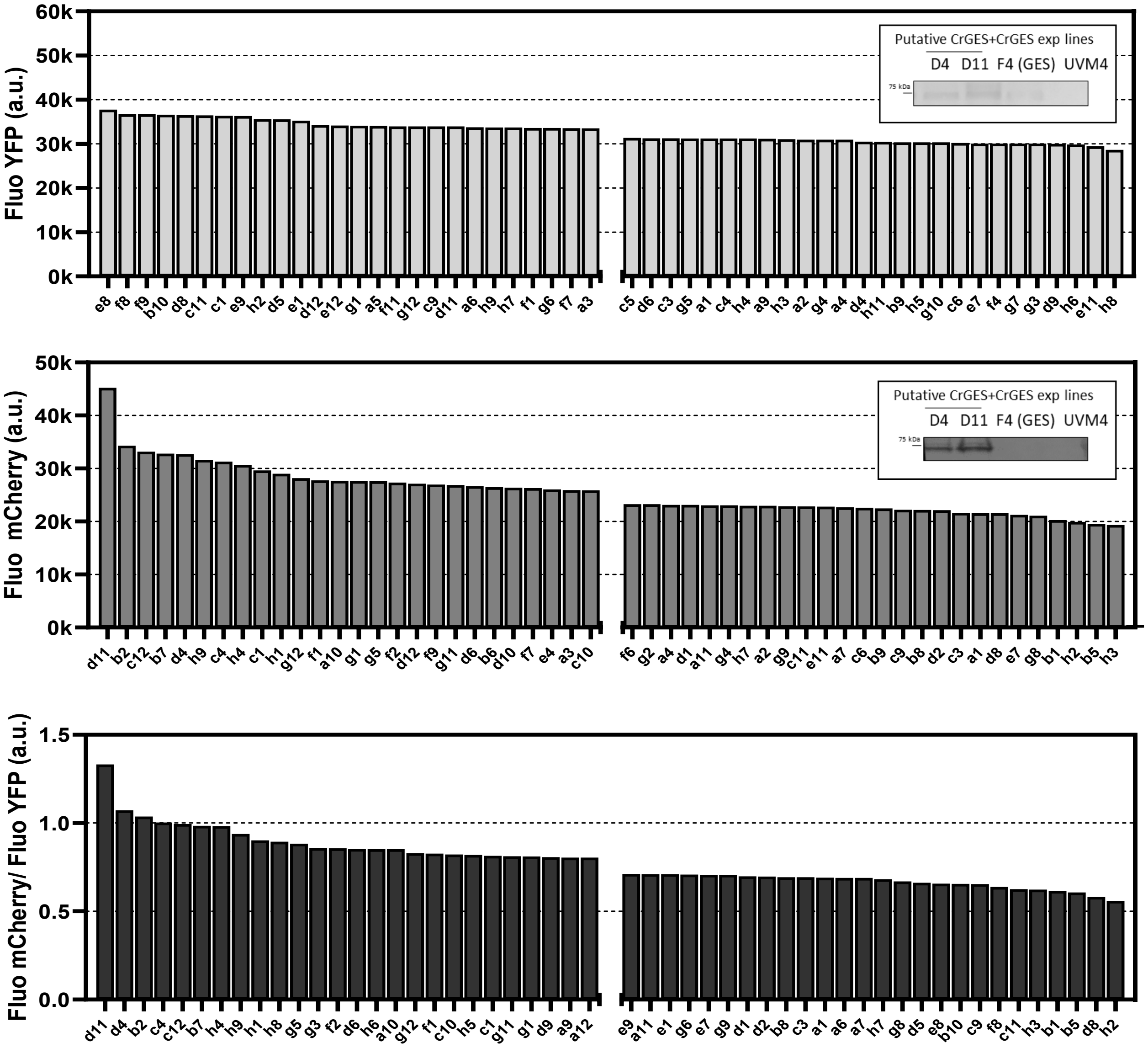

Supplementary Figure 13. *Le*GPPS expressing lines screening. Fluorescence screening of putative lines expressing *Le*GPPS used for protein localization. Lines are decreasingly ordered with a 40-line. Western blot confirming the presence of the protein in the best five pre-screened lines is reported in the insert. For western blot total proteins of 1x10[6] cells were loaded.

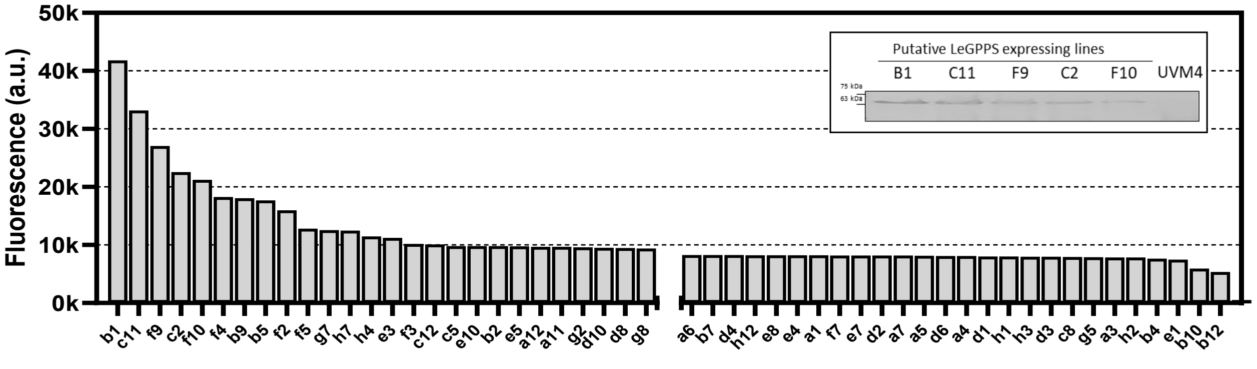

Supplementary Figure 14. *Cr*GES+ *Le*GPPS expressing lines screening. YFP fluorescence screening of putative lines expressing *Cr*GES (F4 background) and *Le*GPPS. Lines are decreasingly ordered with a 40-line. Western blot (against YFP) confirming the presence of both proteins in the best five pre-screened lines is reported in the insert. Total proteins of 1x10[6] cells were loaded and f4 background was used as control both for fluorescence and WB.

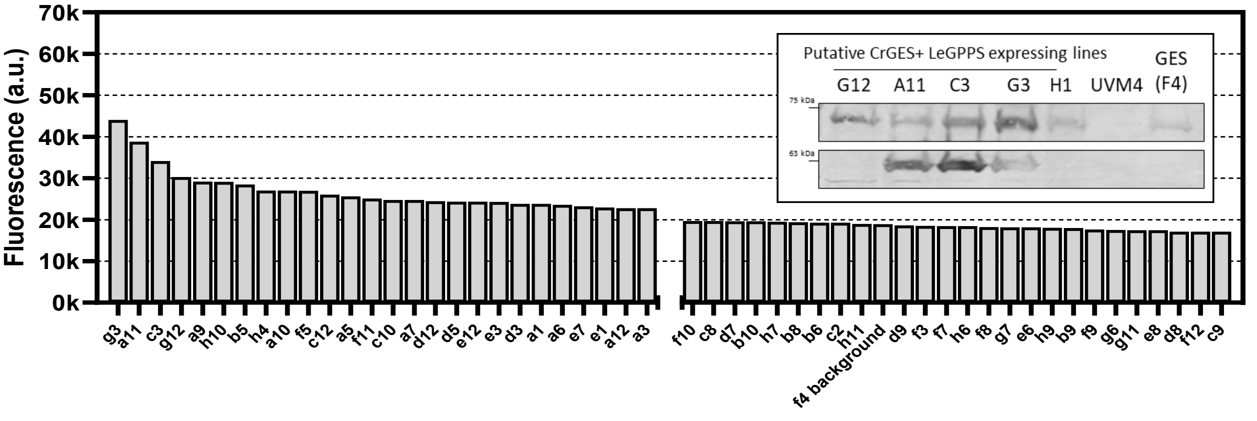

Supplementary Figure 15. *Cr*GES-43aa + *Le*GPPS expressing lines screening. (a) Fluorescence screening of putative lines expressing *Cr*GES-43aa and *Le*GPPS. YFP signal on top, mCherry in the middle, and mCherry/YFP ratio on bottom. Lines are decreasingly ordered with a 40-line. Western blot confirming the presence of both proteins in the best eight pre-screened lines is reported in the insert (anti-YFP on top and anti-mCherry in the middle). For western blot total proteins of 1x10^6 cells were loaded. (b) confocal microscopy localization of *Cr*GES-43aa_YFP and *Le*GPPS_mCherry. Excitation for YFP was 514 nm, 543 nm for mCherry, and 633 nm for chlorophylls. Emission was detected at 522–572 nm for YFP, 560-620 nm for mCherry, and 670–690 nm for chlorophylls. The scale bar represents 5 μm.

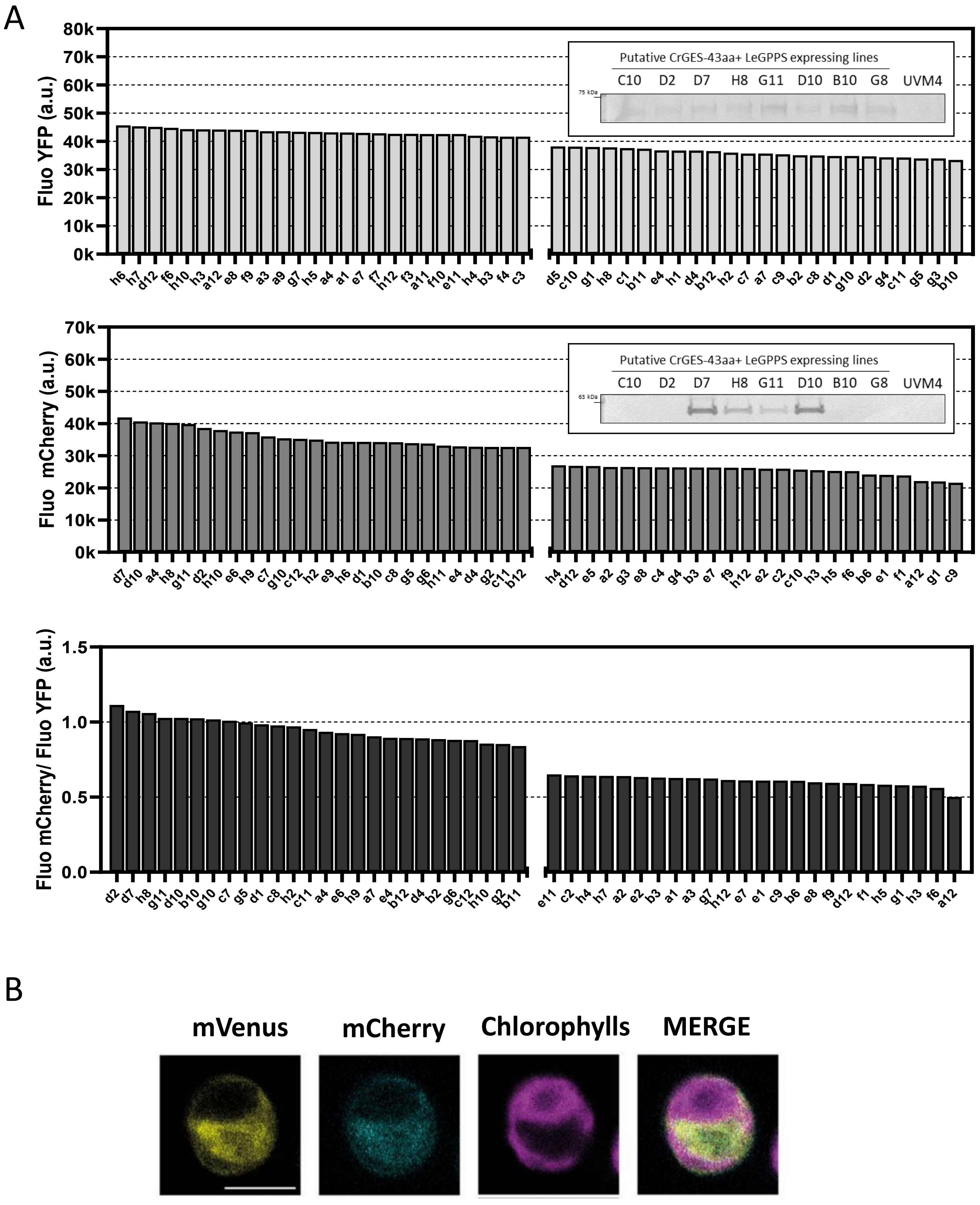

Supplementary Figure 16. *Cr*GES+ PsaD_*Le*GPPS expressing lines screening. Fluorescence screening of putative lines expressing *Cr*GES (F4 background) and *Le*GPPS. YFP signal on top, mCherry in the middle, and mCherry/YFP ratio on bottom. Lines are decreasingly ordered with a 40-line. Western blot confirming the presence of both proteins in the best eight pre-screened lines is reported in the insert (anti-YFP on top and anti-mCherry in the middle). For western blot total proteins of 1x10[6] cells were loaded.

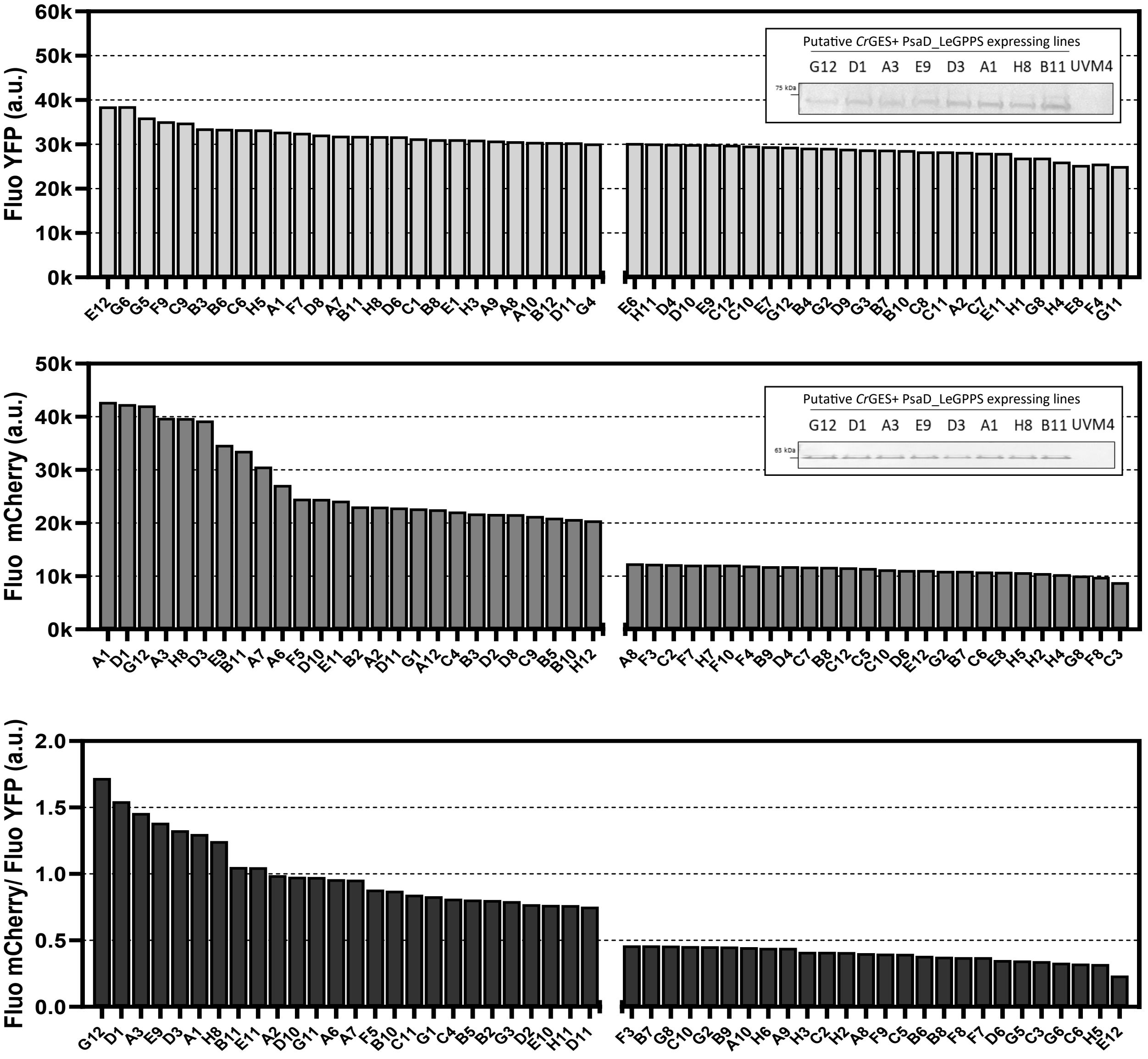

Supplementary Figure 17. *Cr*GES_LeGPPS_YFP screening and analysis. (a) scheme of *Cr*_GES_LeGPPS_YFP vector used (b) Fluorescence screening of putative lines expressing *Cr*GES_LeGPPS_YFP. Lines are decreasingly ordered with a 40-line. Western blot confirming the presence of protein for the best three pre-screened lines is reported in the insert. For western blot total proteins of 1x10^6 cells were loaded. (c) confocal microscopy localization of *Cr*GES_LeGPPS_YFP. Excitation for YFP was 514 nm and 633 nm for chlorophylls. Emission was detected at 522–572 nm for YFP, and 670–690 nm for chlorophylls. The scale bar represents 5 µm. (d) Geraniol accumulation in growth medium for GES_YFP, GES + PsaD_GPPS, and GES_GPPS_YFP expressing lines 47h after inoculum. UVM4 is shown as the background negative control.

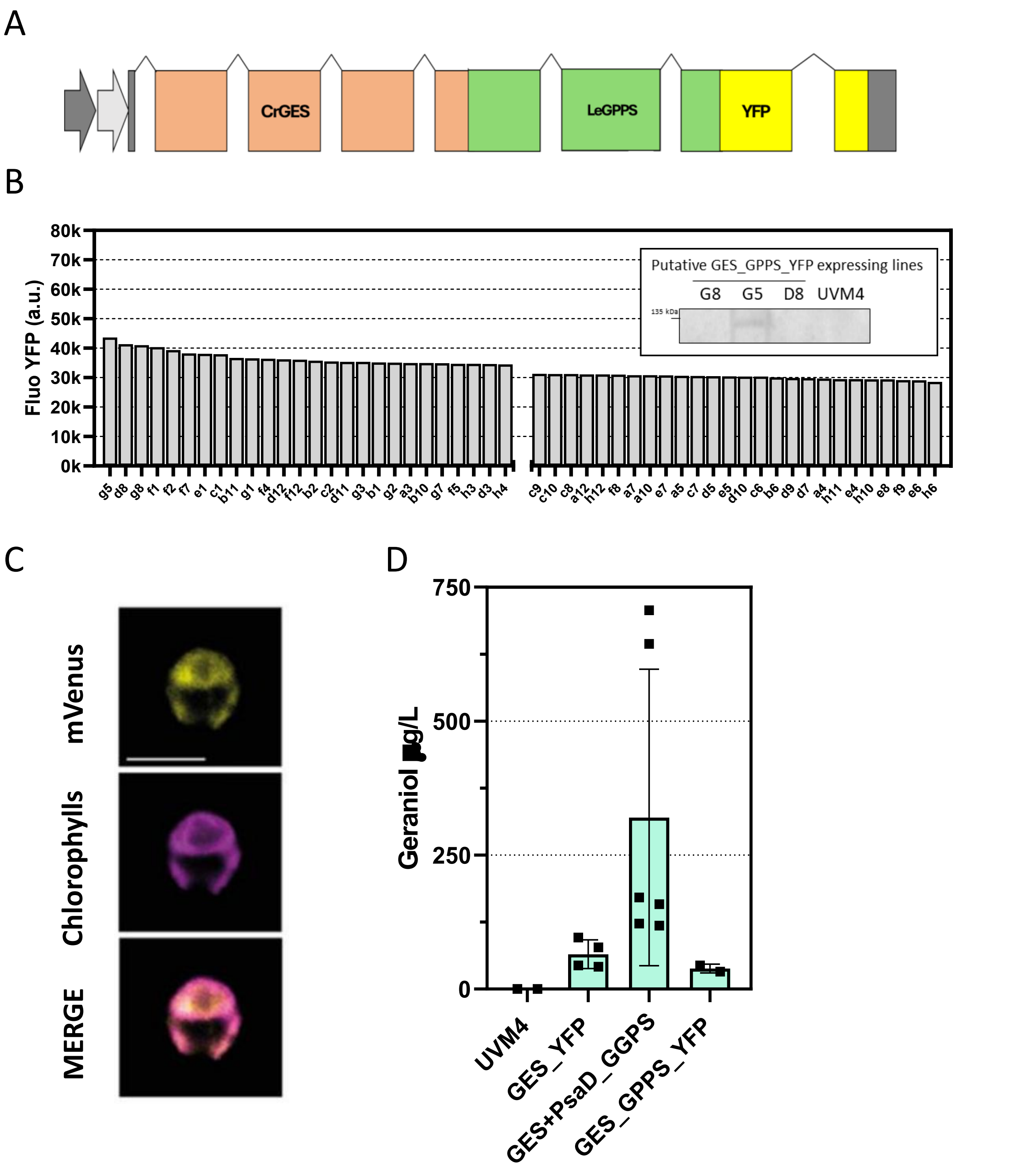

.

Supplementary Figure 18. PsaD_SpDXS expressing lines screening. Fluorescence screening of putative lines expressing PsaD_SpDXS used for protein localization. Lines are decreasingly ordered with a 40-line. Western blot confirming the presence of the protein in the best pre-screened line is reported in the insert. For western blot total proteins of 1x10^6 cells were loaded.

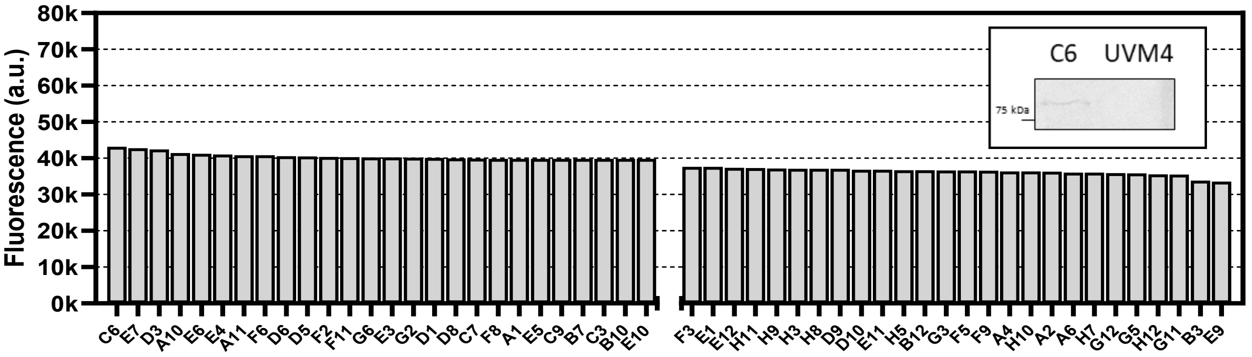

Supplementary Figure 19. Triple-expressing lines geraniol accumulation. Lines expressing *Cr*GES, *Le*GPPS and *Sp*DXS were tested for geraniol production and compared with the double expressing A3 line.

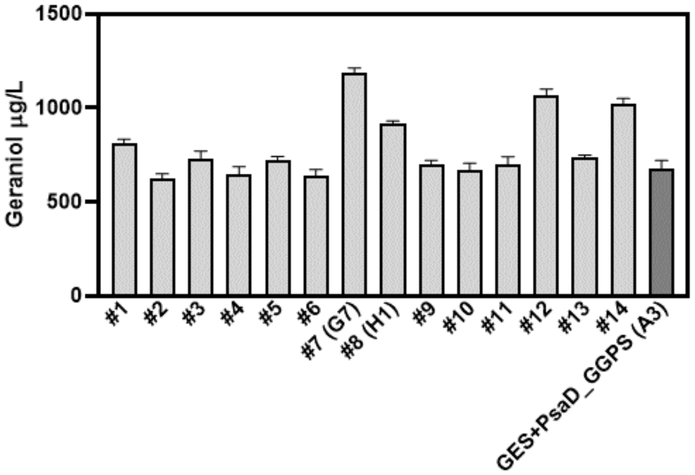

Supplementary Figure 20. Triple-expressing lines growth test. Growth parameters for *Cr*GES+LeGPPS+SpDXS (G7) and UVM4 lines. Growth test was conducted in mixotrophy (TAP) or autotrophy (HS) in low (80 µmol photons m^{-2} s^{-1}) or high (500 µmol photons m^{-2} s^{-1}) light. 720nm optical density and dry biomass weight are shown. Values are the average of 3 independent experiments.

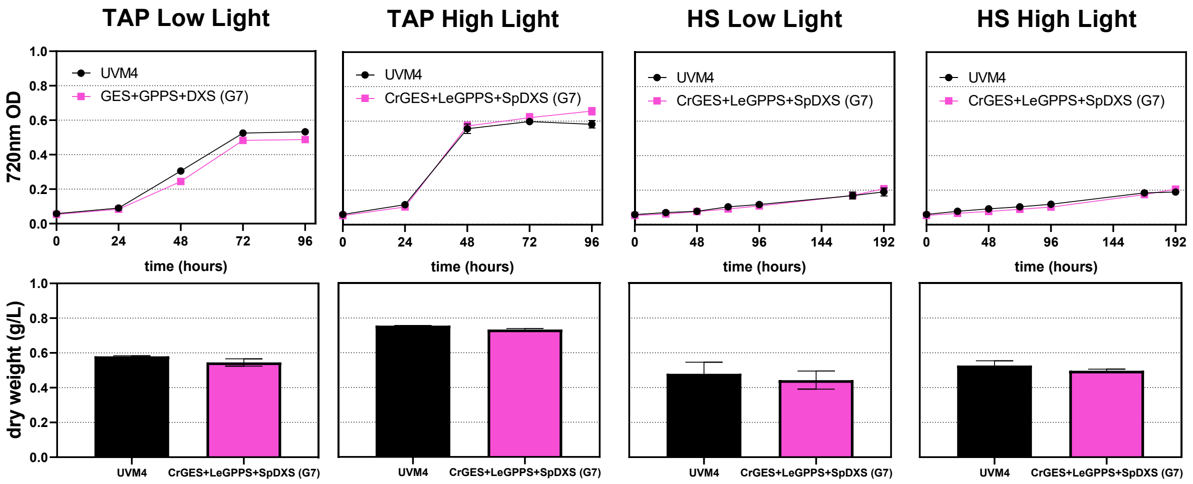

Supplementary Figure 21. Triple-expressing lines pigment analysis. Chlorophyll and carotenoid content of *Cr*GES+LeGPPS+SpDXS (G7) expressing line and UVM4. All parameters were evaluated in low (80 µmol photons m^{-2} s^{-1}) or high (500 µmol photons m^{-2} s^{-1}) light and reported as an average of 3 biological replicates. The error bar represents the standard deviation.

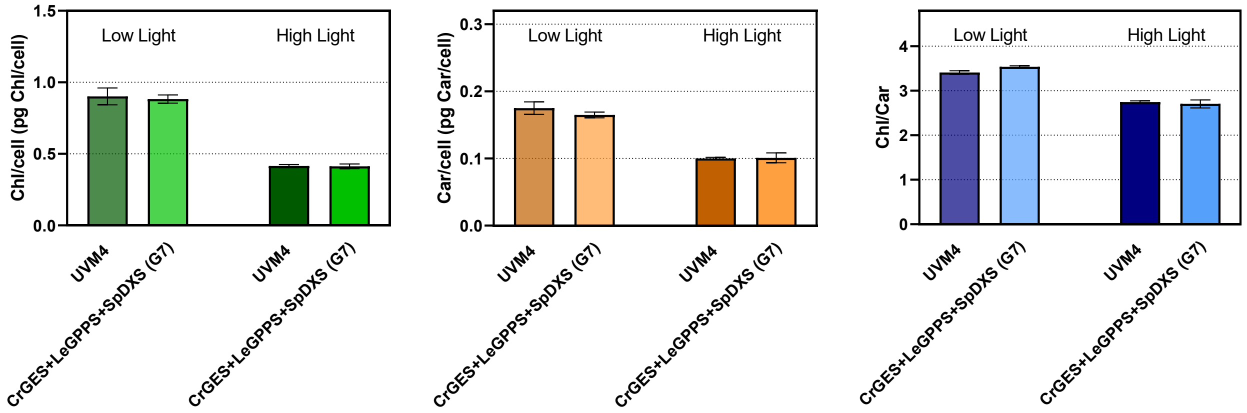

Supplementary Figure 22. Protein sequence of enzymes used for overexpression. Transit peptide, as well as fluorophore sequences, are missing and described in supplementary Figure 2

Catharanthus roseus GES (UniProt:J9PZR5)

MAATISNLSFLAKSRALSRPSSSSLSWLERPKTSSTICMSMPSSSSSSSSSSMSLPLATPLIKDNESLIKFLRQPLVLPH
EVDDSTKRRELLERTRKELELNAEKPLEALKMIDIIQRLGLSYHFEDDINSILTGFSNISSQTHEDLLTASLCFRLLRHN
GHKINPDIFQKFMDNNGKFKDSLKDDTLGMLSLYEASYLGANGEEILMEAQEFTKTHLKNSLPAMAPSLSKKVSQALEQP
RHRRMLRLEARRFIEEYGAENDHNPDLLELAKLDYNKVQSLHQMELSEITRWWKQLGLVDKLTFARDRPLECFLWTVGLL
PEPKYSGCRIELAKTIAILLVIDDIFDTHGTLDELLLFTNAIKRWDLEAMEDLPEYMRICYMALYNTTNEICYKVLKENG
WSVLPYLKATWIDMIEGFMVEAEWFNSDYVPNMEEYVENGVRTAGSYMALVHLFFLIGQGVTEDNVKLLIKPYPKLFSSS
GRILRLWDDLGTAKEEQERGDLASSIQLFMREKEIKSEEEGRKGILEIIENLWKELNGELVYREEMPLAIIKTAFNMARA
SQVVYQHEEDTYFSSVDNYVKALFFTPCF

Lithospermum erythrorhizon GPPS (NCBI:BBG62184.1)

MASQAGAVSLNQKQKFMDVYTVLKSELVKDTLFEWTDDSRQWVERMLDYNVPGGKLNRGLAVIDSYNLLQEGRDLTEDEL
FLASVLGWCVEWFQAYFLVHDDIMDNSTTRRGQPCWYKLPKVGMIAVNDGVILRNHIPRILKKYFRDKPYYVNLLDVFNE
IEFLTASGQMIDLVTTLQGEKDLSKYTLDLYRRIVLFKTSYYSFYLPVACALLMAGEKLEDHDLVKDALIKMGEYFQIQD
DYLDCYGAPETIGKIGTDIEDFKCSWMVVKAVEKCNEGQRKILYENYGMENHASVAKVKALYNDLDLKGVFEEYESSVYK
KLTATIEAHSSPAVQAVLKSFLSKIYKREK

Salvia pomifera DXS partial (NCBI:AXL65958)

SSSAVLPMIKKHKLGVVAALQQDNTNEVVASGGESLTTTTRHKTRALSFTGEKPPTPILDTINYPIHMKNLSVEELERLA
DELREEIVYTVSKTGGHLSSSLGVAELTVALHHVFNTPDDKIIWDVGHQAYPHKILTGRRARMHTIRQTFGLAGFPKRDE
SAHDAFGAGHSSTSISAGLGMAVGRDLLHKDNHVISVIGDGAMTAGQAYEALNNAGFLDSNLIIVLNDNKQVSLPTATVD
GPAPPVGLSKALTKLQASRKFRLLREAANGMTRQMGDQAHEIASKVDTYVKGMMGKPGASLFEELGIYYIGPVDGHNIED
LVYIFKKVKMPAPGPVLIHIITEKGKGYPPAEVAADKMHGVVKFDPTTGKQLKSKTKTKSYTQYFAESLVAEAEQDEKIV
AIHAAMGGGTGLNYFQKRFPDRCFDVGIAEQHAVTFAAGLATEGLKPFCTIYSSFLQRGYDQVVHDVDLQKLPVRFMMDR
AGVVGADGPTHCGAFDTTYMACLPNMVVMAPSDELQLMHMIATAAAIDDRPSCVRYPRGNGIGAPLPPNNKGTPLEVGKG
ILREGSRVAILGFGTIVQNCLAAAQLLEEHGVSVTVADARFCKPLDGDLIKKLVQEHEVLITVEEGSIGGFSAHISHFLS
LNGLLDGNLKWRPMVLPDRYIDHGAQTDQIEEAGLSPKHIAKTVVSLIGGGKDTTLHLINNLGSGSGS

Supplementary Table 1. Construct characteristics. Sum up of construct used in this work. The main characteristics of genetic sequence, as well as protein produced, are listed.

Construct name	Expressed protein	Fluorophore	Gene length with introns (bp)	Predicted MW (with TP)	Localization	Target peptide used
CrGES_YFP	CrGES	YFP	3272	97.5 kDa	Chloro + Chl/Cyt interphase	Native
PsaD_CrGES_YFP	PsaD_CrGES	YFP	3377	101.2 kDa	Chloro	PsaD
PsaD_CrGES-43_YFP	PsaD_CrGES-43aa	YFP	3257	96.7 kDa	Chloro	PsaD
CrGES-43_YFP	CrGES-43aa	YFP	3143	93 kDa	Cyto	/
43aaCrGES_YFP	43aaCrGES	YFP	1184	33.8 kDa	Chl/Cyt interphase	Native (predicted)
60aaCrGES_YFP	60aaCrGES	YFP	1241	35.6 kDa	Chl/Cyt interphase	Native (60aa)
100aaCrGES_YFP	100aaCrGES	YFP	1355	40.2 kDa	Chloro	Native (100aa)
CrGES_mCherry	CrGES	mCherry	3075	97 kDa	Chloro+ Chl/Cyt interphase	Native
LeGPPS_YFP	LeGPPS	YFP	2410	70.2 kDa	Cyto	Native
PsaD_LeGPPS_mCherry	LeGPPS	mCherry	2322	73.3 kDa	Chloro	PsaD
PsaD_SpDXS_CFP_Specto	SpDXS	CFP	3922	110 kDa	Chloro	PsaD
PsaD_SpDXS_MinS	SpDXS	none	3994	112.7 kDa	Chloro	PsaD
