# Supplementary material for: Sustainable Production of Bio-Based Geraniol: Heterologous Expression of Early Terpenoid Pathway Enzymes in Chlamydomonas reinhardtii
Source: ACS Synth Biol. 2025 Aug 26;14(9):3753–66. doi: 10.1021/acssynbio.5c00510 (PMC12455652; doi:10.1021/acssynbio.5c00510)

Sustainable production of bio-based geraniol: heterologous expression of early terpenoid pathway enzymes in *Chlamydomonas reinhardtii*

Federico Perozeni[1#], Edoardo Ceschi[1#], Giovanni Luzzini[1], Davide Slaghenaufi[1], Matteo Pivato[1], Stefano Cazzaniga[1], Thomas Baier[2], Alexander Einhaus[2], Sebastian Overmans[3], Kyle J. Lauersen[3], Maurizio Ugliano[1], Matteo Ballottari[1*]

[1] *Department of Biotechnology, University of Verona, Strada le Grazie 15, 37134 Verona, Italy*

[2] *Bielefeld University, Faculty of Biology, Center for Biotechnology (CeBiTec), Universitätsstrasse 27, 33615, Bielefeld, Germany.*

[3] *Bioengineering Program, Biological Environmental Sciences and Engineering Division (BESE), King Abdullah University of Science and Technology (KAUST), 239555, Thuwal, Saudi Arabia.*

[‡]These authors contributed equally.

[*]**Corresponding author:** Matteo Ballottari

Email: matteo.ballottari@univr.it

Phone: +39 045 8027823

Supplementary File 2

Complete maps of the main plasmids used in this work for the expression in *Chlamydomonas reinhardtii*. Detailed sequences are available as a Supplementary file 1 in .txt format, compatible with Snapgene or similar software.

pOpt2_GES_YFP_Paro

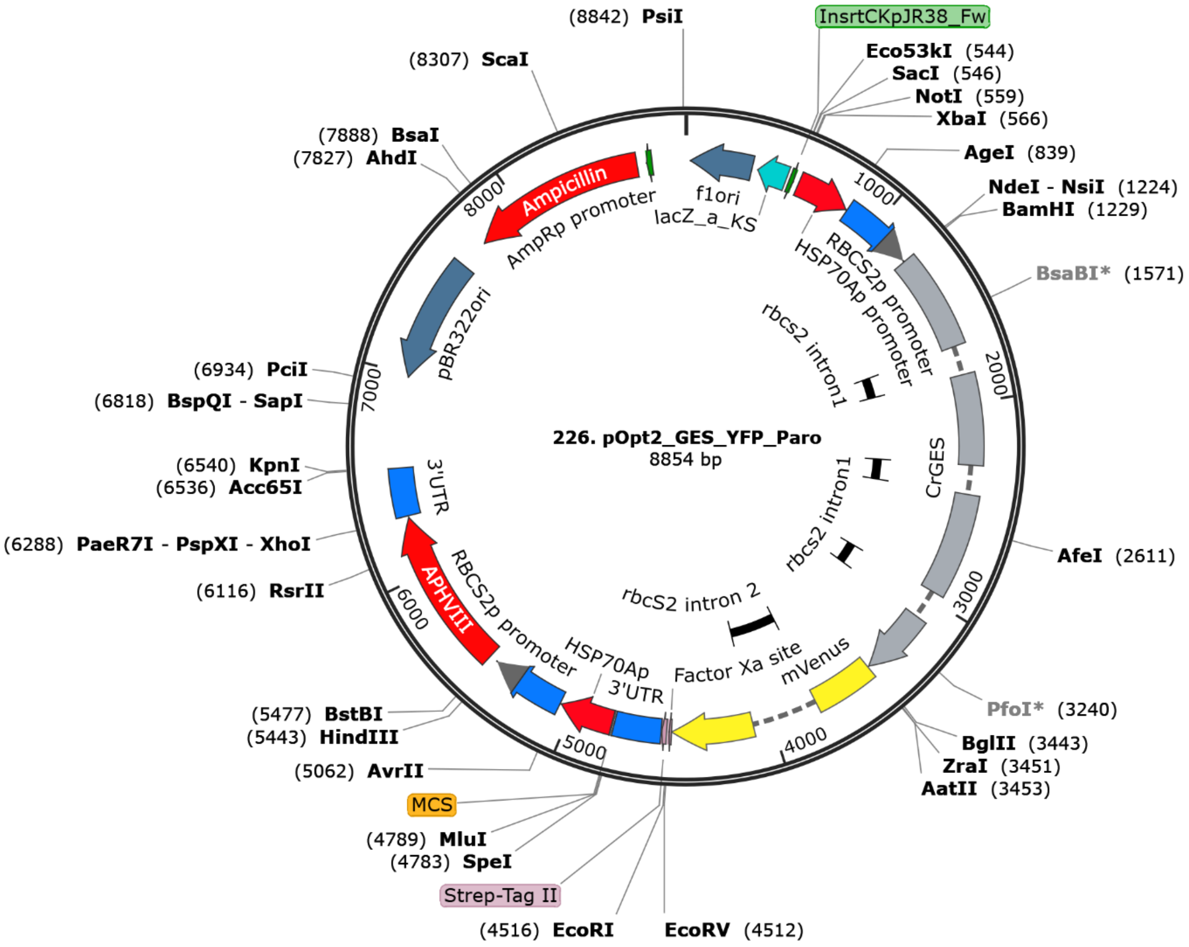

pOpt2_PsaD_LeGPPS_mCherry_Paro

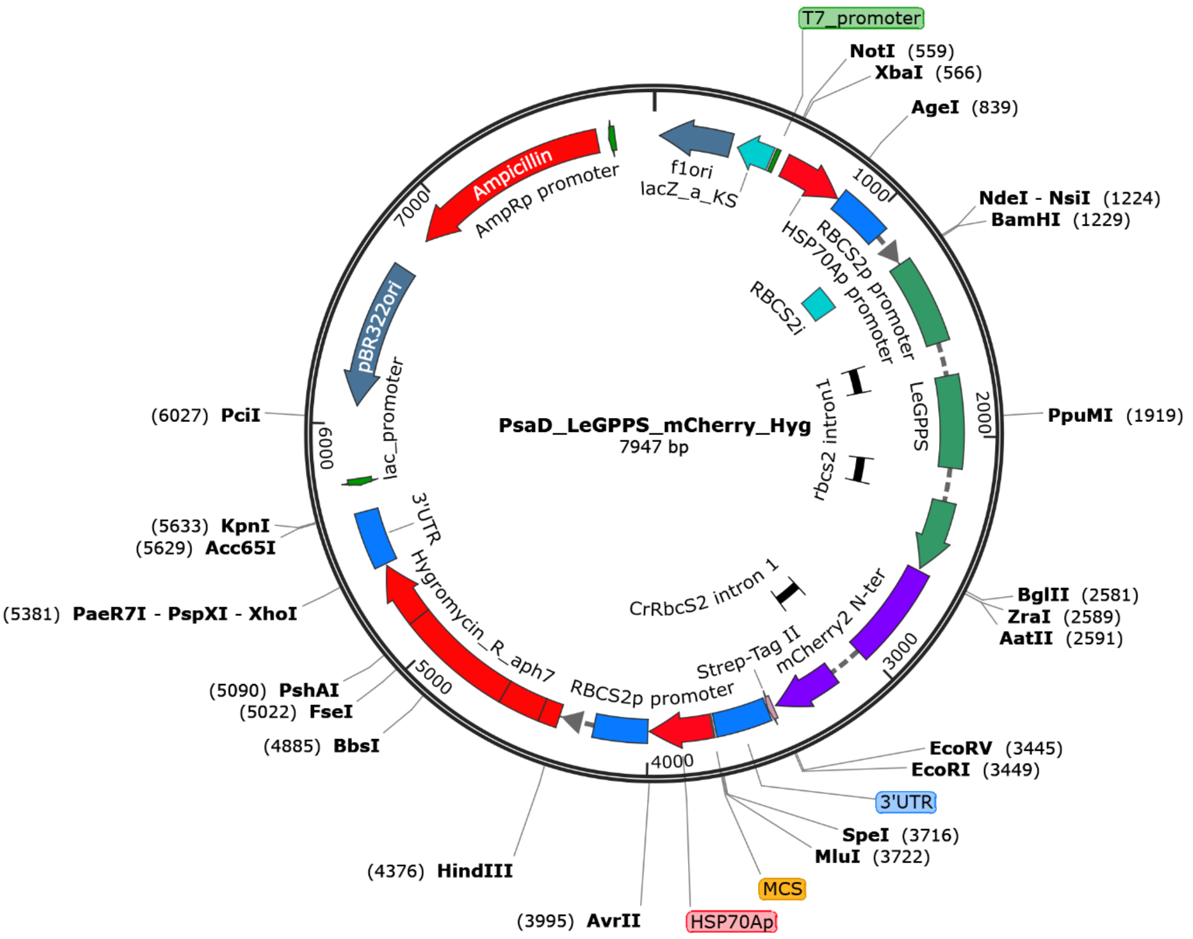

pOpt2_PsaD_SpDXS_CFP_Specto

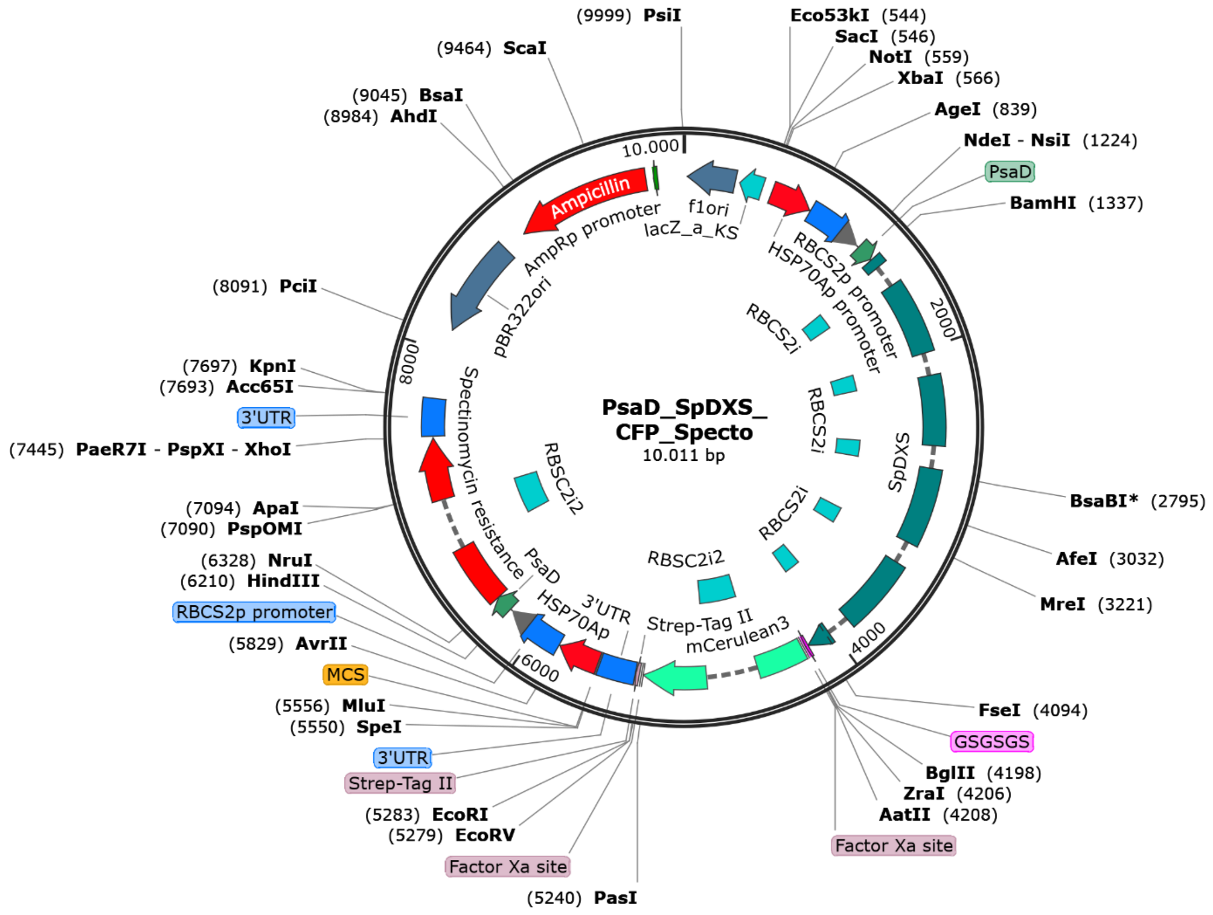

pOpt2_PsaD_SpDXS_MinS

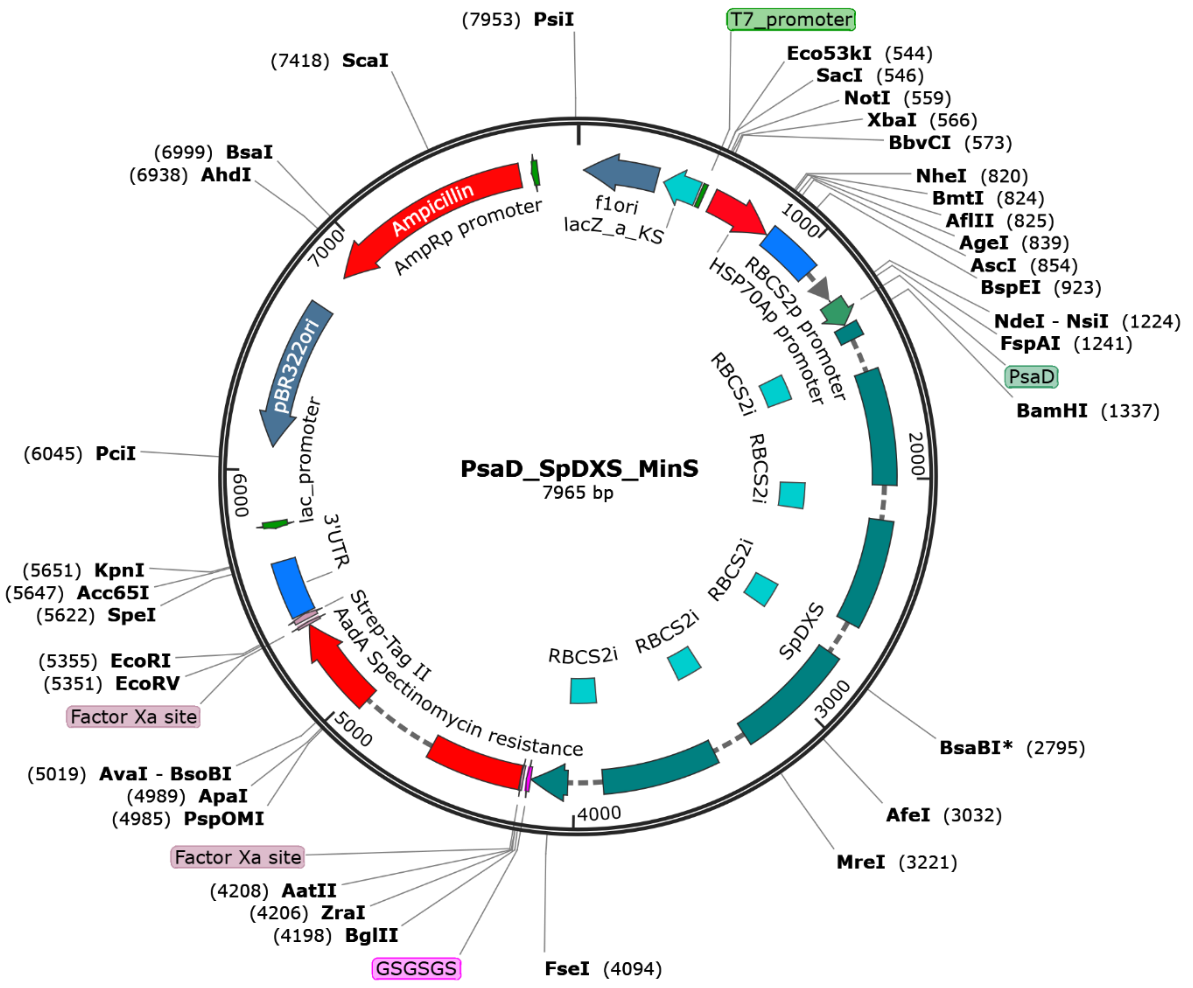

Supplement: Supplementary file 3 [file sb5c00510_si_003.pdf]
